# Supplementary material for: Graphene enhances artemisinin production in the traditional medicinal plant Artemisia annua via dynamic physiological processes and miRNA regulation
Source: Plant Commun. 2023 Nov 2;5(3):100742. doi: 10.1016/j.xplc.2023.100742 (PMC10943550; doi:10.1016/j.xplc.2023.100742)
Supplement: Document S1. Supplemental Figures 1–15 and Supplemental Tables 1–3 [file mmc1.pdf]

Plant Communications, Volume *5*

Supplemental information

Graphene enhances artemisinin production in the traditional medicinal plant *Artemisia annua* via dynamic physiological processes and miRNA regulation

Junfeng Cao, Zhiwen Chen, Luyao Wang, Ning Yan, Jialing Lin, Lipan Hou, Yongyan Zhao, Chaochen Huang, Tingting Wen, Chenyi Li, Saeed ur Rahman, Zehui Liu, Jun Qiao, Jianguo Zhao, Jie Wang, Yannan Shi, Wei Qin, Tong Si, Yuliang Wang, and Kexuan Tang

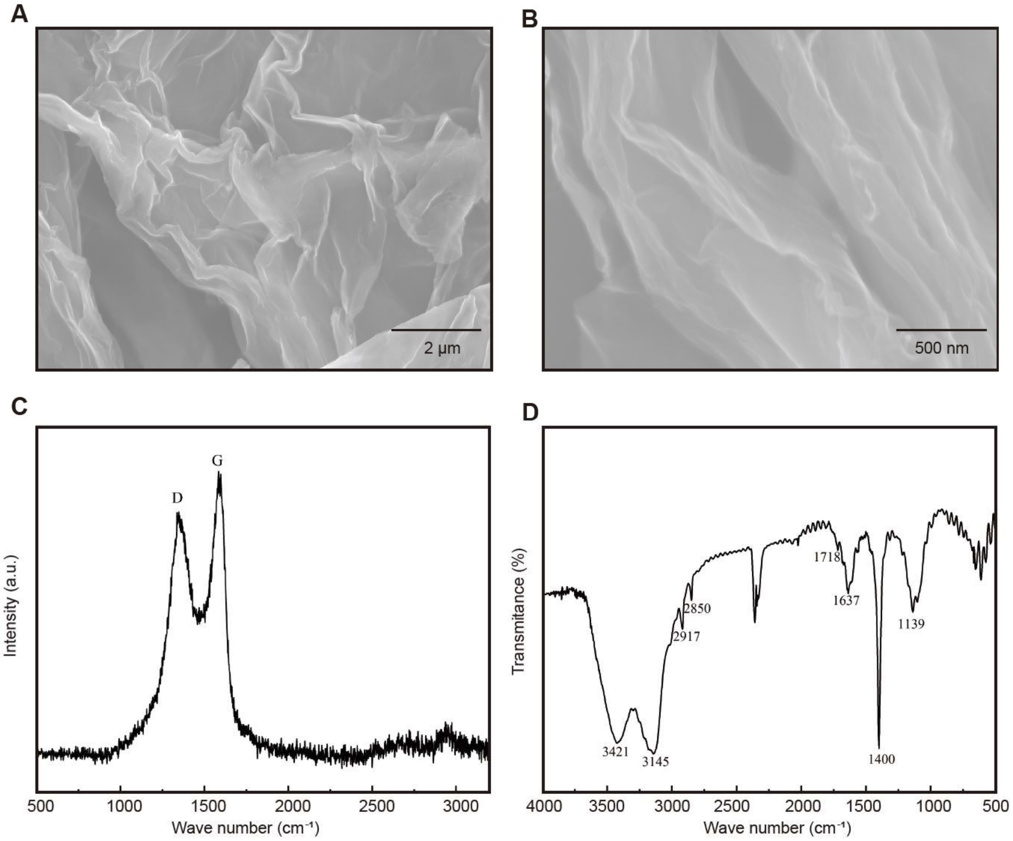

Supplementary Figure 1 Characterization of graphene.

(A and B) SEM image, **(C)** Raman spectrum, and **(D)** Fourier transform infrared (FT-IR) spectrum.

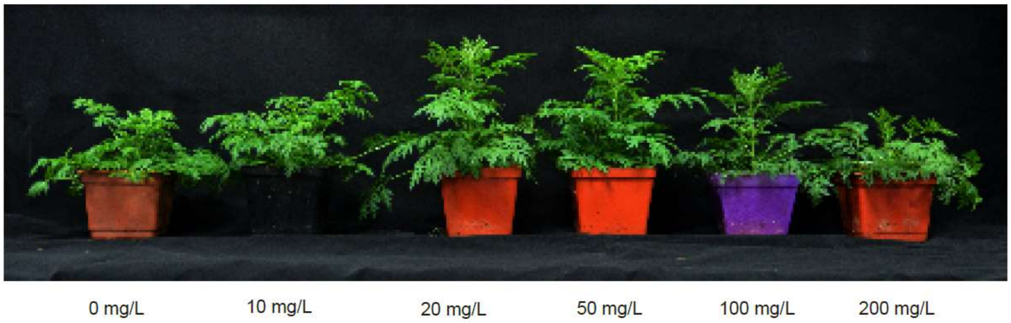

Supplementary Figure 2 Phenotypes of *A. annua* treated with different concentration of graphene.

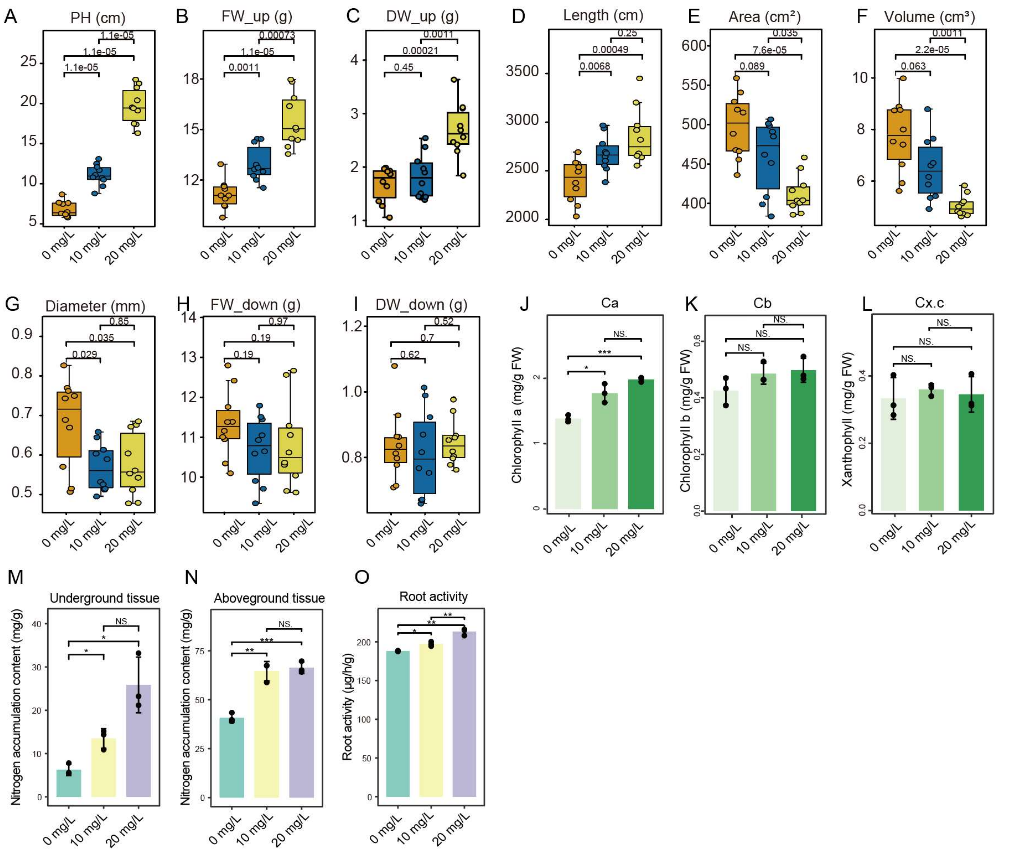

Supplementary Figure 3 Statistics of phenotypes and physiological data of *A. annua* treated with graphene.

(A-C) statistics analysis of overground parts seedlings exposed to different graphene concentrations shown in **Figure 1A**. **(A)** Plant height (PH). **(B)** Fresh weight of shoots (FW_up). **(c)** Dry weight of shoots (DW_up), mean ± SD, n = 10, Student's *t*-test.

(D-I) Statistics of root phenotypes generated from **Figure 1B**, including total root length **(D)**, total root surface area **(E)**, total root volume **(F)** and root average diameter **(G)**. **(H)** Fresh weight of roots (FW_down). **(I)** Dry weight of roots (DW_down), mean ± SD, n = 10, Student's *t*-test.

(J-L) Statistics of the photosynthetic pigments **(J)** chlorophyll a, **(K)** chlorophyll b and **(L)** xanthophyll.

(M-O) Statistics of nitrogen absorption and assimilation in **Figure 1**. **(M and N)** total nitrogen accumulated in the underground **(M)** and aboveground **(N)** parts of the seedlings. **(O)** root activity measured by TTC.

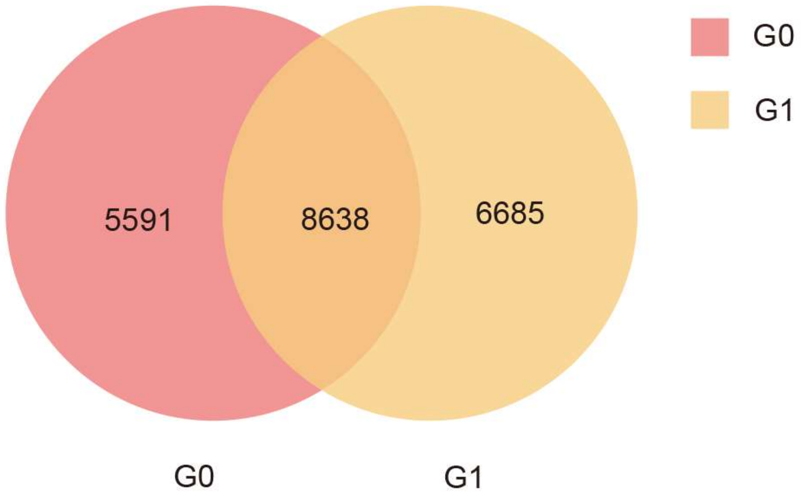

Supplementary Figure 4 Differentially expressed genes of leaf between CK and graphene treatment groups. G0, 0 vs 10 mg/L; G1, 0 vs 20 mg/L.

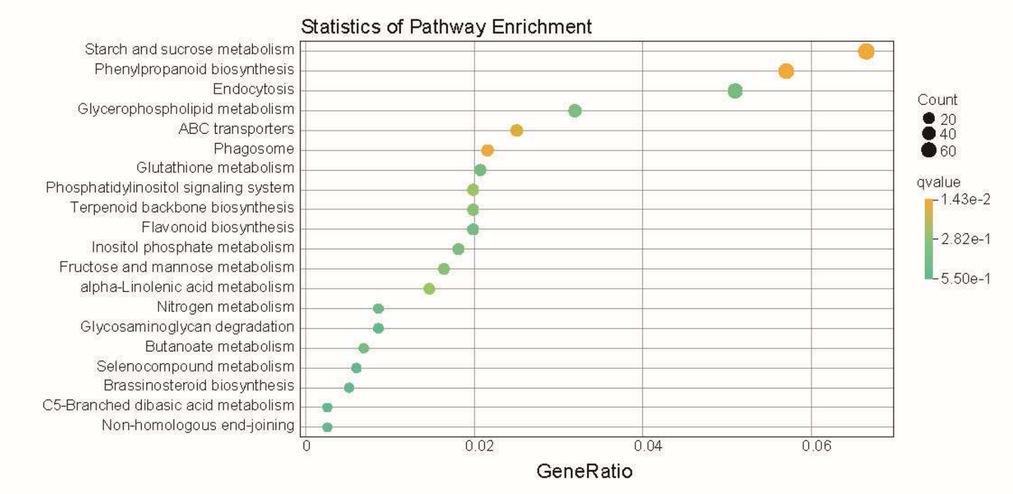

Supplementary Figure 5 Top 20 KEGG pathway analyses of the enriched differentially expressed genes after graphene treatment.

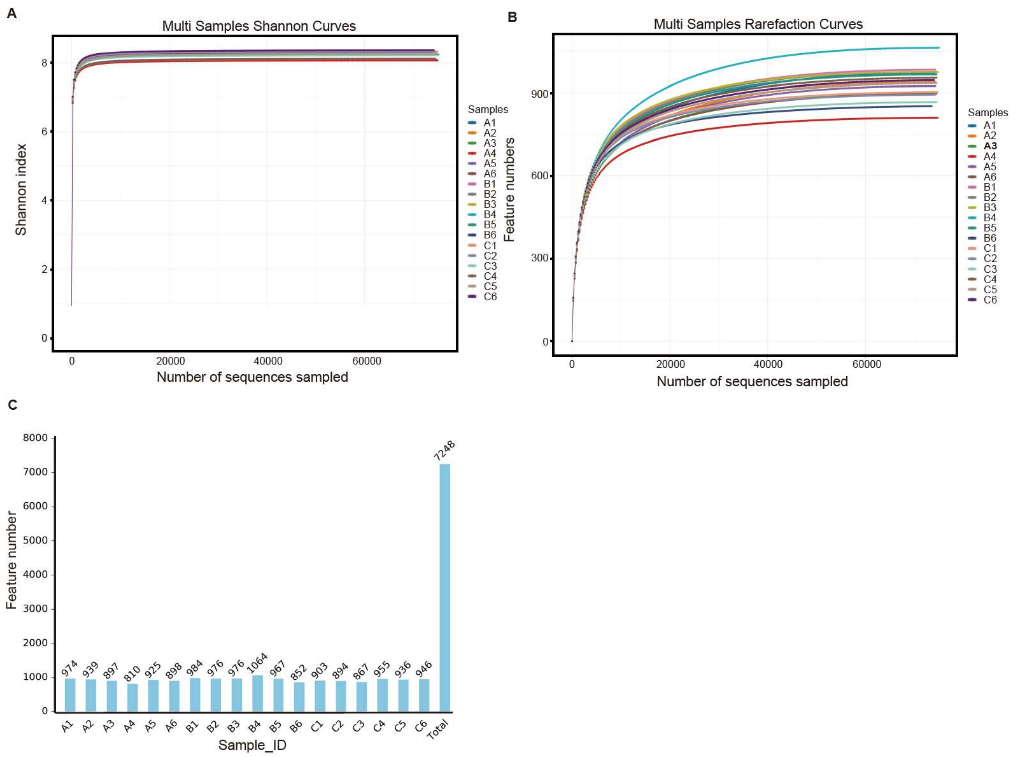

Supplementary Figure 6 Sequencing statistics and basic OTU analyses.

(A) bacterial 16S DNA Shannon-Wiener curve, **(B)** bacterial 16S DNA Rarefaction curve, **(C)** bacterial species OTUs number. Group A: 0 mg/L, Group B: 10 mg/L, Group C: 20 mg/L

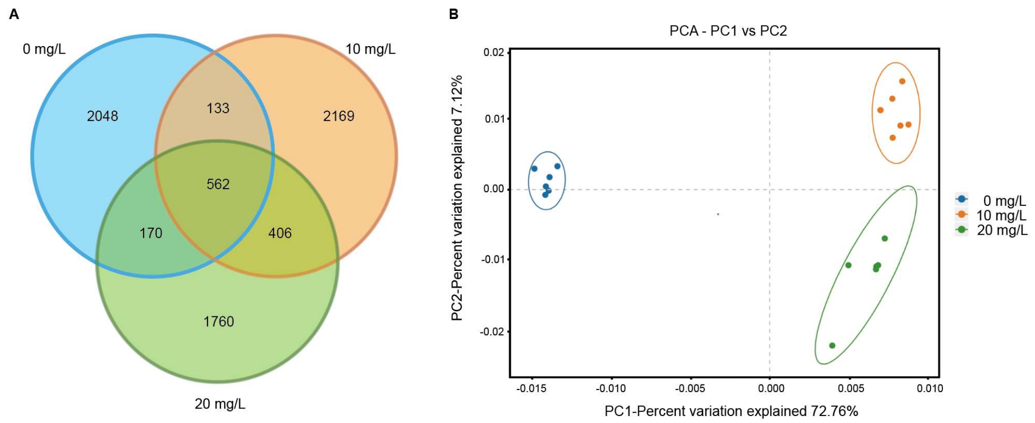

Supplementary Figure 7 Rhizosphere microbial changes between CK and graphene treatment groups.

(A) Venn diagrams of the bacterial OTUs number, **(B)** Principal Component Analysis (PCA) of the rhizosphere bacterial community.

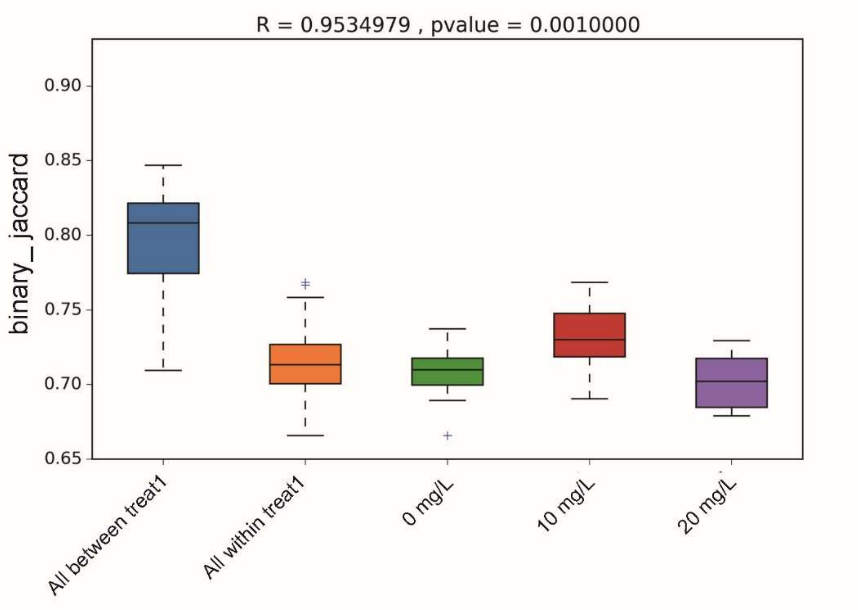

Supplementary Figure 8 ANOSIM analysis boxplot.

The R-value (between -1 and 1) is greater than 0, indicating that the difference between the groups is significant. The credibility of the statistical analysis is expressed by *P*-value, and *P* <0.05 indicates significance.

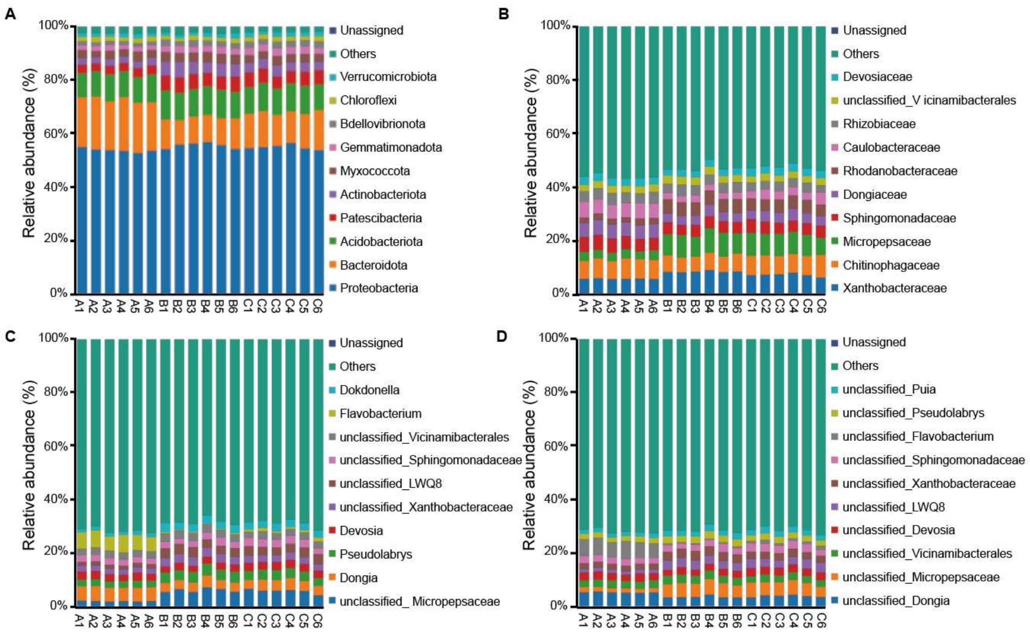

Supplementary Figure 9 Changes in rhizosphere bacterial community diversity between the control (A samples) and graphene treatment groups (B samples 10 mg/L, C samples 20 mg/L). Proportion (%) of major bacteria at the phylum **(A)**, family **(B)**, genus **(C)** and species **(D)** levels.

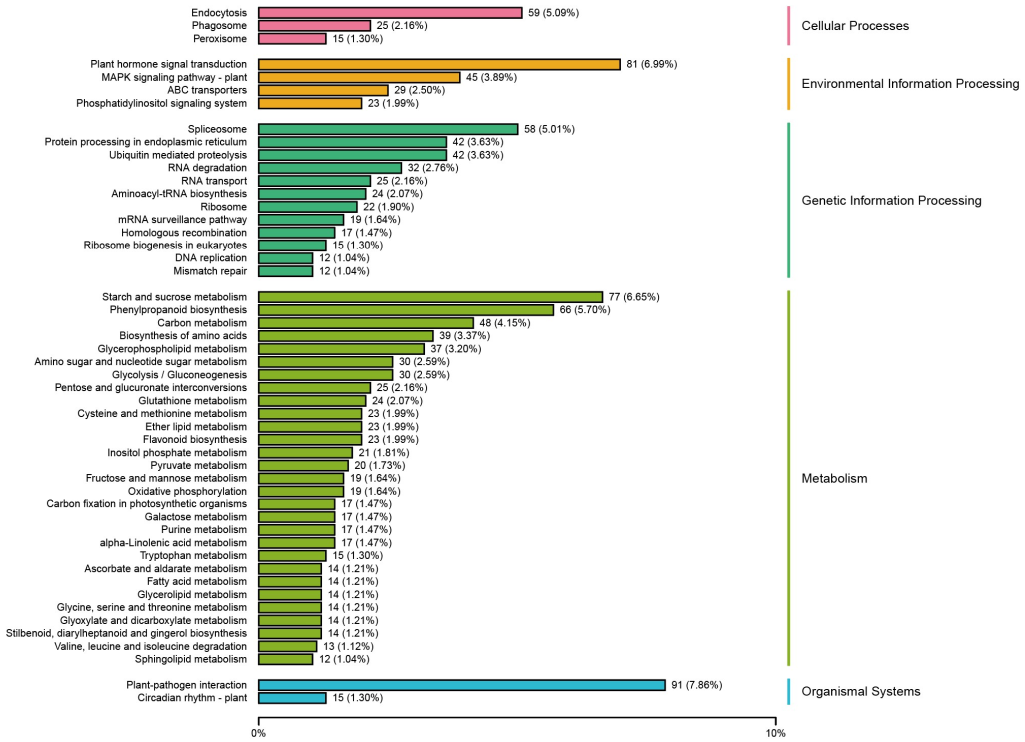

Supplementary Figure 10 KEGG classification analyses of the enriched differentially expressed genes.

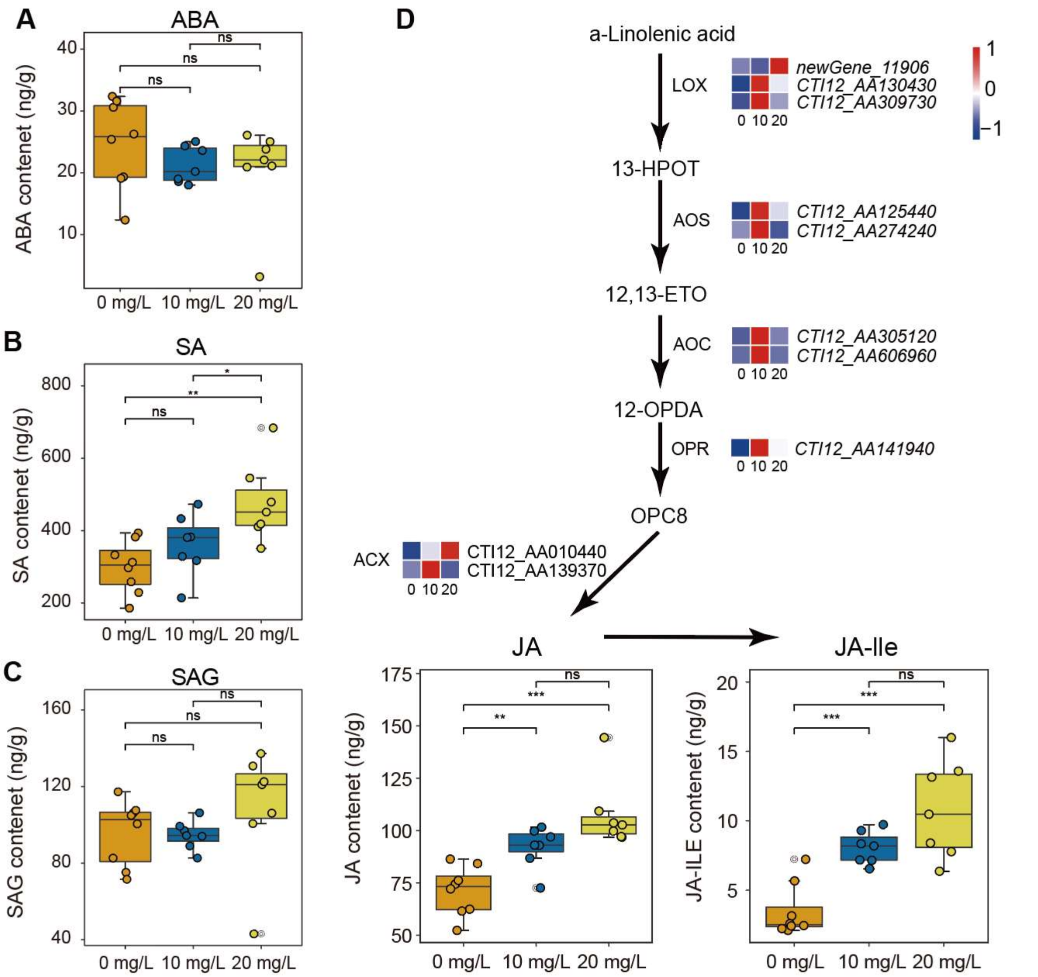

Supplementary Figure 11 Graphene promotes the biosynthesis of stress-related phytohormones

(A-D) The contents of endogenous ABA **(A)**, SA **(B)** and its derivative SAG **(C)** with graphene treatments. **(D)** The enhanced biosynthetic pathway and products of JA, the essential regulator for artemisinin. The expression level of enzymes located in JA pathway is presented by the heatmap according to the RNA-seq. The contents of endogenous JA and JA-IIE are under the pathway, mean ± SD, n = 7, **P* < 0.05, ***P* < 0.01, Student's *t*-test.

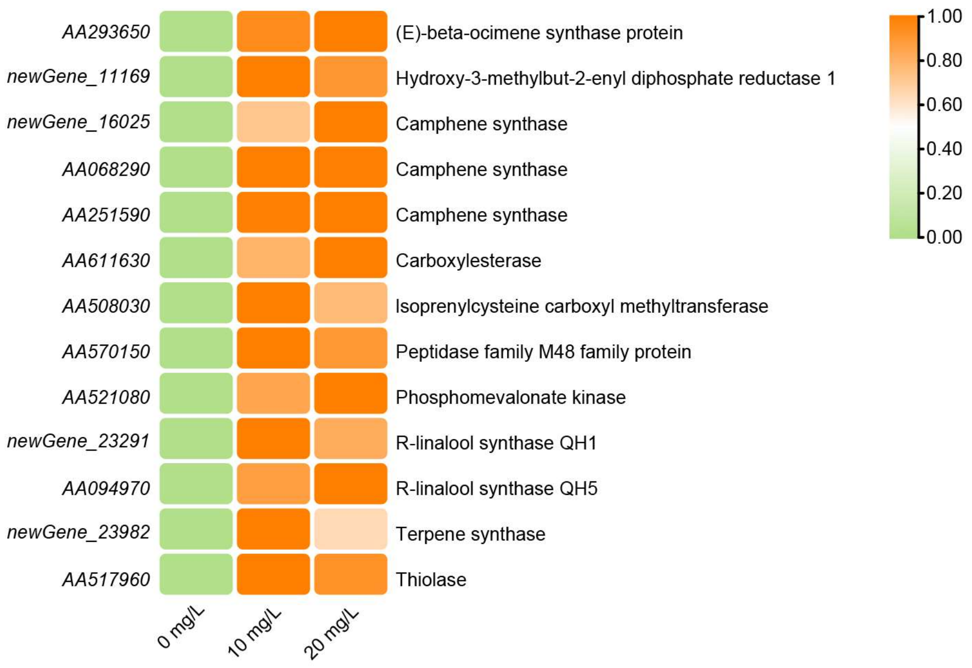

Supplementary Figure 12 Heatmap of DEGs involved in terpenoid backbone biosynthesis.

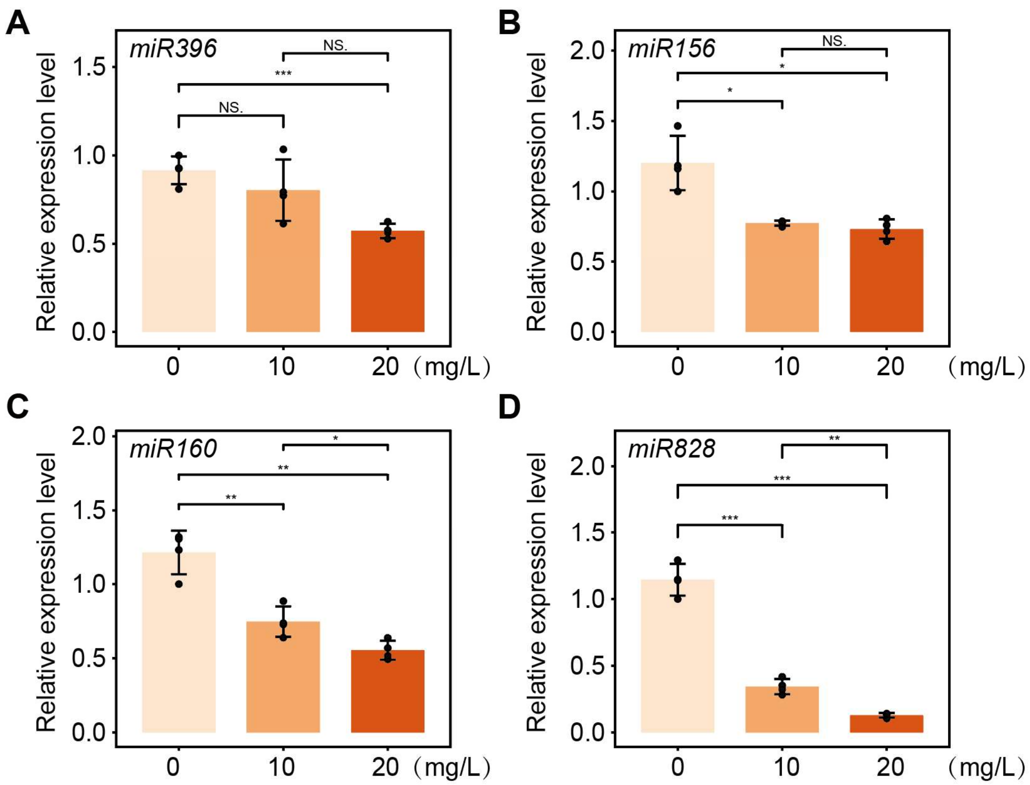

Supplementary Figure 13 Graphene decreases the expression of miRNAs.

(A-D) Relative expression of mature miRNAs in *A. annua*, the level at 0 mg/L was set to one (means of triplicates ± s.d.), U6 served as internal reference. **P* < 0.05, ***P* < 0.01, ****P* < 0.001, Student's *t*-test

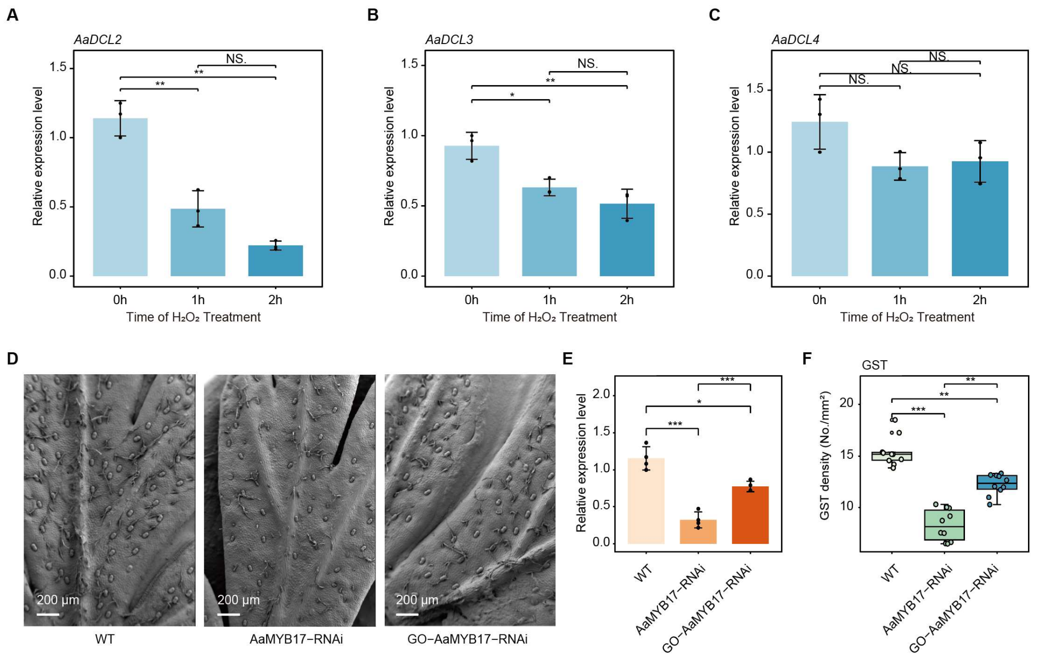

Supplementary Figure 14 Graphene blocks the siRNA biogenesis to rescue the RNAi effect through dicers.

(A-C) Relative expression of Dicers in *A. annua* treated with 1 mM H_2O_2. The level at 0 h (untreated) was set to one (means of triplicates ± s.d.). *$P < 0.05$, **$P < 0.01$, Student's *t*-test.

(H) Image of GSTs on leaves from wild type (WT), AaMYB17 RNAi lines and 20 mg/L graphene treated AaMYB17 RNAi lines (GPE-AaMYB17-RNAi). **(I)** Relative expression of AaMYB17 in leaves as shown in **(H)** (mean ± s.d., n=4, *$P < 0.05$, ***$P < 0.001$, Student's *t*-test). **(J)** GST densities of the samples from **(H)** (mean ± s.d., n=10, **$P < 0.01$, ***$P < 0.001$, Student's *t*-test).

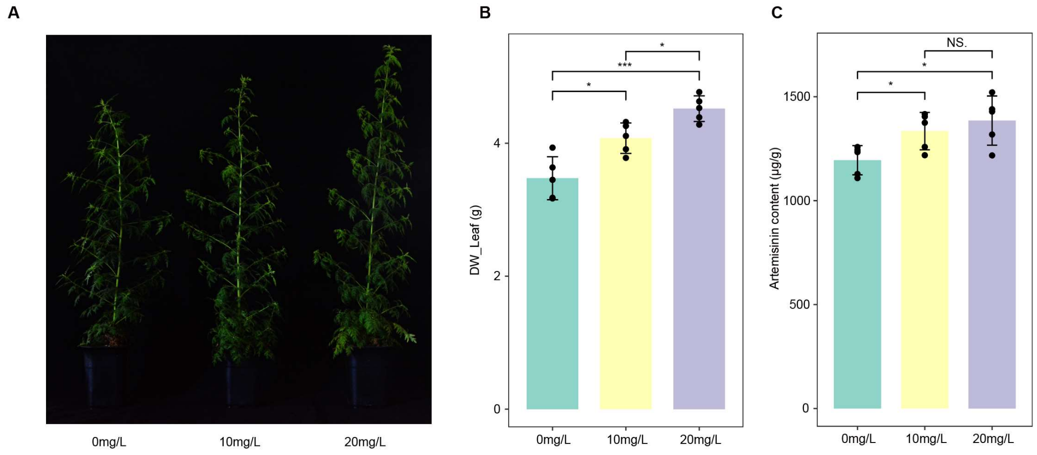

Supplementary Figure 15 Graphene improves artemisinin yield

(A) Image of 4-month-old plants treated with graphene. **(B)** the dry weight of total leaves. **(C)** the artemisinin content per unit weight of total leaves from **(A)** mean ± s.d., n=5, **P* < 0.05, ****P* < 0.001, Student's *t*-test

Supplementary Table 1. Statistics of bacteria 16S DNA sequencing data.

Sample ID	Raw Reads	Clean Reads	Denoised Reads	Merged Reads	Non-chimeric Reads	Effective Ratio (%)
A0-1	80,009	79,674	77,884	75,467	74,381	92.97%
A0-2	80,256	79,889	77,986	75,411	74,277	92.55%
A0-3	80,157	79,820	78,166	75,791	74,739	93.24%
A0-4	80,065	79,664	78,064	75,938	74,818	93.45%
A0-5	80,070	79,706	77,881	75,447	74,300	92.79%
A0-6	80,020	79,619	77,866	75,471	74,290	92.84%
B10-1	80,246	79,921	78,076	75,348	74,485	92.82%
B10-2	80,053	79,729	78,083	75,622	74,674	93.28%
B10-3	79,946	79,614	77,966	75,778	74,778	93.54%
B10-4	80,023	79,681	78,051	76,124	75,233	94.01%
B10-5	79,730	79,401	77,784	75,463	74,686	93.67%
B10-6	80,161	79,782	77,834	75,446	73,645	91.87%
C20-1	79,973	79,616	77,833	75,725	74,901	93.66%
C20-2	79,994	79,643	77,823	75,221	74,317	92.90%
C20-3	80,107	79,772	78,009	75,643	74,642	93.18%
C20-4	79,987	79,654	77,912	75,638	74,684	93.37%
C20-5	80,118	79,761	77,984	75,518	74,720	93.26%
C20-6	80,085	79,728	77,794	75,512	74,204	92.66%

Group A: 0 mg/L, Group B: 10 mg/L, Group C: 20 mg/L

Supplementary Table 2. Statistics of the bacterial OTUs number at different taxonomy levels: kingdom, phylum, class, order, family, genus and species.

Sample	Kingdom	Phylum	Class	Order	Family	Genus	Species
A0-1	2	24	47	114	201	294	339
A0-2	1	22	44	108	191	288	331
A0-3	1	21	41	105	188	276	314
A0-4	2	22	44	110	189	282	317
A0-5	1	21	45	115	196	283	327
A0-6	2	22	42	105	185	281	323
B10-1	1	22	46	112	200	289	329
B10-2	1	23	44	114	199	298	330
B10-3	1	23	46	115	207	301	333
B10-4	1	23	48	113	193	294	335
B10-5	1	23	46	117	206	295	323
B10-6	1	24	49	118	200	290	318
C20-1	1	21	45	113	201	296	324
C20-2	1	21	43	108	195	286	318
C20-3	1	21	43	112	195	289	313
C20-4	1	24	50	119	206	303	341
C20-5	1	20	43	107	194	288	314
C20-6	1	19	42	110	194	293	327
Total	2	29	69	167	317	527	633

Group A: 0 mg/L, Group B: 10 mg/L, Group C: 20 mg/L

Supplementary Table 3. Major primers used in this investigation

Primer ID	Sequence	Purpose
ADS-RT-F	AATGGGCAAATGAGGGACAC	qRT-PCR
ADS-RT-R	TTTCAAGGCTCGATGAACTATG	qRT-PCR
CYP71AV1-RT-F	CGAGACTTTAACTGGTGAGATTGT	qRT-PCR
CYP71AV1-RT-R	CGAAGCGACTGAAATGACTTTACT	qRT-PCR
DBR2-RT-F	GCGGTGGTTACACTAGAGAACTT	qRT-PCR
DBR2-RT-R	ATAATCAAAACTAGAGGAGTGACCC	qRT-PCR
ALDH1-RT-F	GGACTTGCCTCAGGTGTAT	qRT-PCR
ALDH1-RT-R	GTGCCTCTAATCCTTGTTC	qRT-PCR
AaMYB17-RT-F	TCATTACTACTCCCATCACCCAAC	qRT-PCR
AaMYB17-RT-R	CATCGCTCCTTCTGGCACAT	qRT-PCR
AaGSW2-RT-F	TTCCTCATCTTCAACTTCACCAC	qRT-PCR
AaGSW2-RT-R	CGCTACTCGTGGGAGAAACAT	qRT-PCR
AaHD1-RT-F	GCTTGATCCTCACTGCGGTAT	qRT-PCR
AaHD1-RT-R	TACGTTCTGGGACGAGTTGCT	qRT-PCR
AaSPL9-RT-F	TGGAACTCTGTTATCTGCCCGC	qRT-PCR
AaSPL9--RT-R	ATCCTGAACTCCGCTAAAACCTGT	qRT-PCR
actin-RT-F	CCAGGCTGTTCAGTCTCTGTAT	qRT-PCR
actin-RT-R	CGCTCGGTAAGGATCTTCATCA	qRT-PCR
miR828	TCTTGCTCAAATGAGTATTCTA	microRNA qRT-PCR
mir160	TATGAGGAGCCATGCATGTAT	microRNA qRT-PCR
mir156	TGACAGAAGAGAGTGAGCAC	microRNA qRT-PCR
mir396	TTCCACAGCTTTCTTGAACTG	microRNA qRT-PCR
U6	TGTATCGTTCCAATTTTATCGGATGT	microRNA qRT-PCR
