# Supplementary material for: Graphene enhances artemisinin production in the traditional medicinal plant Artemisia annua via dynamic physiological processes and miRNA regulation
Source: Plant Commun. 2023 Nov 2;5(3):100742. doi: 10.1016/j.xplc.2023.100742 (PMC10943550; doi:10.1016/j.xplc.2023.100742)
Supplement: Document S2. Article plus supplemental information [file mmc2.pdf]

Plant Communications
Research article

CellPress Partner Journal

# Graphene enhances artemisinin production in the traditional medicinal plant *Artemisia annua* via dynamic physiological processes and miRNA regulation

Junfeng Cao[1,8], Zhiwen Chen[2,3,8], Luyao Wang[4,5,8], Ning Yan[6], Jialing Lin[3], Lipan Hou[3], Yongyan Zhao[4,5], Chaochen Huang[3], Tingting Wen[3], Chenyi Li[3], Saeed ur Rahman[1], Zehui Liu[2], Jun Qiao[2], Jianguo Zhao[2], Jie Wang[6], Yannan Shi[7], Wei Qin[1], Tong Si[6], Yuliang Wang[1] and Kexuan Tang[1,*]

[1]Frontiers Science Center for Transformative Molecules, Joint International Research Laboratory of Metabolic and Developmental Sciences, Plant Biotechnology Research Center, Fudan-SJTU Nottingham Plant Biotechnology R&D Center, School of Agriculture and Biology, Shanghai Jiao Tong University, Shanghai 200240, China

[2]Engineering Research Center of Coal-based Ecological Carbon Sequestration Technology of the Ministry of Education, Key Laboratory of Graphene Forestry Application of National Forest and Grass Administration, Shanxi Datong University, Datong 037009, China

[3]National Key Laboratory of Plant Molecular Genetics, Institute of Plant Physiology and Ecology/CAS Center for Excellence in Molecular Plant Sciences, Chinese Academy of Sciences, Shanghai 200032, China

[4]Hainan Institute, Zhejiang University, Yongyou Industry Park, Yazhou Bay Sci-Tech City, Sanya 572000, China

[5]College of Agriculture and Biotechnology, Zhejiang University, Hangzhou 310058, China

[6]Shandong Provincial Key Laboratory of Dryland Farming Technology, College of Agronomy, Qingdao Agricultural University, Qingdao 266109, China

[7]Institute of Millet Crops, Hebei Academy of Agriculture & Forestry Sciences/Hebei Branch of China National Sorghum Improvement Center, Shijiazhuang 050035, China

[8]These authors contributed equally to this article.

***Correspondence: Kexuan Tang (kxtang@sjtu.edu.cn)**

https://doi.org/10.1016/j.xplc.2023.100742

## ABSTRACT

**We investigated the effects of graphene on the model herb *Artemisia annua*, which is renowned for producing artemisinin, a widely used pharmacological compound. Seedling growth and biomass were promoted when *A. annua* was cultivated with low concentrations of graphene, an effect which was attributed to a 1.4-fold increase in nitrogen uptake, a 15%–22% increase in chlorophyll fluorescence, and greater abundance of carbon cycling–related bacteria. Exposure to 10 or 20 mg/L graphene resulted in a ~60% increase in $H_2O_2$, and graphene could act as a catalyst accelerator, leading to a 9-fold increase in catalase (CAT) activity *in vitro* and thereby maintaining reactive oxygen species (ROS) homeostasis. Importantly, graphene exposure led to an 80% increase in the density of glandular secreting trichomes (GSTs), in which artemisinin is biosynthesized and stored. This contributed to a 5% increase in artemisinin content in mature leaves. Interestingly, expression of *miR828* was reduced by both graphene and $H_2O_2$ treatments, resulting in induction of its target gene *AaMYB17*, a positive regulator of GST initiation. Subsequent molecular and genetic assays showed that graphene-induced $H_2O_2$ inhibits micro-RNA (miRNA) biogenesis through Dicers and regulates the miR828–AaMYB17 module, thus affecting GST density. Our results suggest that graphene may contribute to yield improvement in *A. annua* via dynamic physiological processes together with miRNA regulation, and it may thus represent a new cultivation strategy for increasing yield capacity through nanobiotechnology.**

**Key words:** *Artemisia annua*, artemisinin, glandular secreting trichomes GSTs, miRNA, graphene

**Cao J., Chen Z., Wang L., Yan N., Lin J., Hou L., Zhao Y., Huang C., Wen T., Li C., Rahman S., Liu Z., Qiao J., Zhao J., Wang J., Shi Y., Qin W., Si T., Wang Y., and Tang K.** (2024). Graphene enhances artemisinin production in the traditional medicinal plant *Artemisia annua* via dynamic physiological processes and miRNA regulation. Plant Comm. **5**, 100742.

Published by the Plant Communications Shanghai Editorial Office in association with Cell Press, an imprint of Elsevier Inc., on behalf of CSPB and CEMPS, CAS.


© 2023 The Author(s).
This is an open access article under the CC BY license (http://creativecommons.org/licenses/by/4.0/).

## INTRODUCTION

In modern agriculture, the Green Revolution has markedly increased the efficiency of global agricultural production through advanced cultivation and breeding techniques. The application of nanobiotechnology is expected to spur new economic growth in the agricultural field (Wang and White, 2022). In crop cultivation, nanomaterials (NMs) or nanoparticles (NPs) could play roles as fertilizers and pesticides to improve crop nutrition and protection (Kah et al., 2019). For example, $CeO_2$ NPs have been reported to have reactive oxygen species (ROS)-scavenging activity, increasing plant stress tolerance and photosynthesis (Giraldo et al., 2014), and application of nanoscale particles like copper and Ag/Ag-Si can suppress pathogen infection in crops (Borgatta et al., 2018). NM-encapsulated micronutrients may control the release of fertilizer at a suitable time and in appropriate crop tissues, thus enhancing fertilizer efficiency (Li et al., 2023). Furthermore, nanopesticides, such as a layered double hydroxide loaded with double-stranded RNA, can help to deliver biopesticide molecules and prevent their environmental degradation (Jain et al., 2022).

Graphene is classified as a member of the carbon NM family (Chae et al., 2020). This two-dimensional carbon NM possesses numerous favorable properties, including electrical conductivity, strength, thermal conductivity, and light transmittance (Avouris, 2010). Graphene has widespread applications in daily life (Mukherjee et al., 2016; Chen et al., 2022a), and numerous studies have demonstrated its beneficial effects on plant growth processes. The application of 50–200 μg/ml graphene solution to 400 g of soil increased the germination rate of cotton by up to 20% (Pandey et al., 2019). Irrigation with 25 mg/l graphene solution once a week, starting from the sowing stage, enhanced the root growth of 65% of tested angiosperm species (Chen et al., 2022b). Incorporation of 50 g/kg of few-layer graphene into the soil for maize cultivation significantly boosted seedling growth via increases of 80.01%, 69.39%, and 66.67% in N, P, and K uptake, respectively (Wang et al., 2023). Graphene also exhibits antifungal activity against *Bipolaris sorokiniana*. When added to the growth medium at 500 mg/l, it reduced colony size by up to 64% after 7 days (Zhang et al., 2022). In addition, a 0.1 mg/l graphene solution extended the vase life of cut flowers by 1 day (He et al., 2018). Hence, graphene shows significant potential in various agricultural domains. However, its positive effect on the production of medicinal plants remains uncertain.

*Artemisia annua* is one of the most famous traditional Chinese medicinal plants and has been documented and used as a medicine tracing back to 2000 years ago in ancient China (Normile, 2015). Artemisinin, the bioactive compound derived from *A. annua*, is an essential drug for elimination of malaria. Artemisinin-based combination therapies have been recognized as the first choice by the World Health Organization and have saved millions of lives (Zheng et al., 2023). *A. annua* is the only natural source of artemisinin, and its content of this substance is extremely low: about 0.01%–1.0% by dry weight (Hassani et al., 2020). Thus, a number of efforts have been made to improve its production. *A. annua* germplasm has been collected, and a genetic map, metabolomic data, and high-quality genome have been produced to accelerate molecular breeding (Graham et al., 2010; Ma et al., 2015; Shen et al., 2018; Liao et al., 2022). Because metabolic engineering is another key approach used to produce artemisinin, the artemisinin biosynthetic pathway has also been decoded (Hassani et al., 2020). In brief, artemisinin is a sesquiterpene lactone generated from the terpene precursor farnesyl diphosphate (FPP). The rate-limiting enzyme amorpha-4,11-diene synthase converts FPP into amorpha-4,11-diene in the first step of artemisinin biosynthesis. The cytochrome P450 monooxygenase CYP71AV1 then catalyzes the three-step oxidation of amorpha-4,11-diene to artemisinic acid, artemisinic alcohol, and artemisinic aldehyde. Artemisinic aldehydes are used as substrates to produce dihydroartemisinic acid (DHAA) through the catalysis of double-bond reductase 2 and aldehyde dehydrogenase 1. Finally, arteannuin B, derived from artemisinic acid and DHAA, undergoes a light-induced non-enzymatic photochemical oxidation process to generate the final product. During biosynthesis, phytohormones such as jasmonic acid (JA) (Ma et al., 2018) and abscisic acid (ABA) (Zhang et al., 2015; Yuan et al., 2023), as well as the environmental factor light (Liu et al., 2023), act as key regulators to enhance the reaction.

There are two types of trichomes on *A. annua* leaves: nonglandular trichomes (NGTs) and glandular secreting trichomes (GSTs). GSTs are multicellular structures derived from epidermal cells that synthesize, store, and secrete specialized metabolites (Chalvin et al., 2020). GSTs are also the factory for artemisinin production (Hassani et al., 2020). Thus, increasing the density of GSTs is the most effective strategy for enhancing artemisinin yield (Xiao et al., 2016). In *A. annua*, the R2R3-MYB MIXTA1/HD-ZIP IV HD8 complex (Shi et al., 2018; Yan et al., 2018; Xie et al., 2021a) can activate the homeodomain leucine zipper (HD-ZIP) IV factor HD1 to induce GST initiation (Yan et al., 2017). Other R2R3 v-myb avian myeloblastosis viral oncogene homolog (MYB) proteins, including AaMYB17 (Qin et al., 2021) and AaMYB108 (Liu et al., 2023), are also core factors that integrate environmental and phytohormone signals to promote GST growth. Notably, the WRKY transcription factor AaGSW2 could be an alternative dominant factor for GSTs by binding directly to the promoter of HD1 (Xie et al., 2021b). MicroRNAs (miRNAs) have also been identified as indispensable modulators of trichome formation. For NGTs, *miR156*-targeted *SPL9* regulates trichome distribution after bolting (Yu et al., 2010) via crosstalk with the miR171–lost meristems (LOM) module through a protein interaction between SPL9 and LOM (Xue et al., 2014). Constitutive expression of *miR319* promotes trichome initiation in *Populus tomentosa* (Fan et al., 2020) and elongation of fiber trichomes in cotton (Cao et al., 2020). However, few studies have addressed the relationship between miRNAs and GSTs. In *A. annua*, a single study reported that *miR160* reduces the formation of GSTs by targeting and cleaving *AaARF1* (Guo et al., 2022).

At present, technical bottlenecks hinder our ability to increase the yield of artemisinin through traditional cross-breeding and molecular design breeding strategies. To broaden the application of NMs to traditional Chinese herbs and avoid biotechnological barriers, we devised a cost-effective, graphene-based cultivation strategy for *A. annua*. In this work, we used *A. annua* as a model crop for traditional Chinese medicinal plants to assess the capacity of graphene to act as a nanofertilizer. We cultivated *A. annua* seedlings with graphene and comprehensively assessed the environmental, physiological, and molecular effects of the nano-based cultivation strategy.

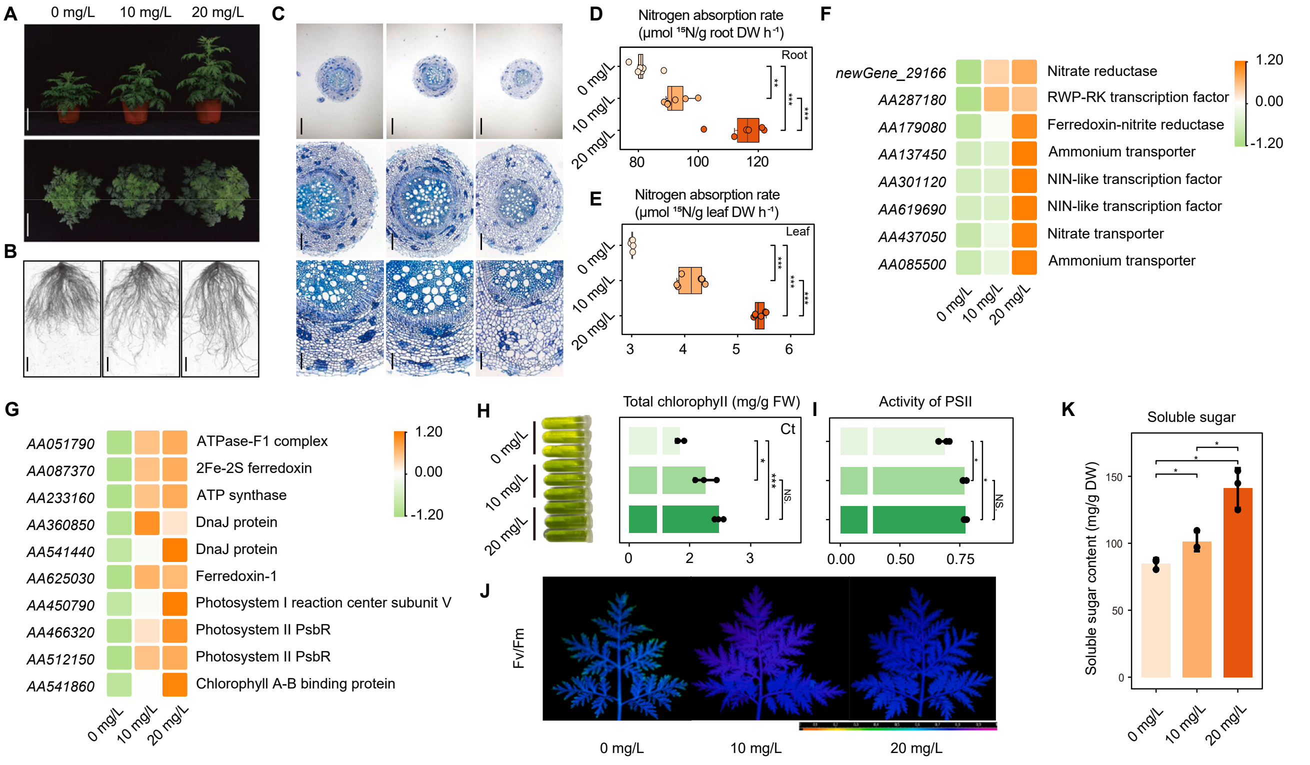


**Figure 1. Graphene promoted growth and induced dynamic physiological processes in *A. annua*.**

**(A–C)** Phenotype analysis of seedlings exposed to different graphene concentrations.

**(A)** Images of shoots from different graphene treatments. Scale bar, 10 cm.

**(B)** Images of root architecture analysis. Scale bar, 5 cm.

**(C)** Root cross-sections. Scale bars: 500 μm (top row), 200 μm (center row), and 100 μm (bottom row).

**(D–F)** Graphene enhanced the absorption, transport, and assimilation of nitrogen.

**(D and E)** Graphene treatment increased rates of $^{15}$N-labeled $^{15}NH_4Cl$ uptake by roots **(D)** and transfer to leaves **(E)**; mean ± SD, n = 6; $^{**}p<0.01$, $^{***}p<0.001$, Student's *t*-test.

**(F)** Heatmap of genes related to nitrogen metabolism and signaling whose expression was induced by graphene.

**(G–K)** Graphene treatment increased photosynthesis.

**(G)** Heatmap of genes related to the photosynthetic system whose expression was induced by graphene.

**(H)** Image of extracted pigments and total chlorophyll contents.

**(I and J)** Maximal photochemical efficiency of photosystem II (PSII) (Fv/Fm). The false color code shown at the bottom of the image **(J)** ranges from 0 (black) to 1 (purple), and Fv/Fm values were calculated **(I)**.

**(K)** Content of soluble sugars; means of triplicates ± SD; $^{*}p<0.05$, $^{***}p<0.001$, Student's *t*-test.

# RESULTS

## Characteristics of graphene NMs

Under scanning electron microscopy (SEM), the graphene used in this research had a stacked and folded appearance, with a clearly layered structure (Supplemental Figure 1A and 1B). Raman spectroscopy revealed the representative D and G peaks of graphene (Supplemental Figure 1C). The infrared spectrum was used to characterize the surface oxygen-containing functional groups of graphene, including C-O (1139 $cm^{-1}$), C-OH (1400 $cm^{-1}$), C=O (1718 $cm^{-1}$), O-H (3145 $cm^{-1}$), and -OH (3421 $cm^{-1}$) (Supplemental Figure 1D).

## Graphene promoted the growth and development of *A. annua* seedlings

We performed initial growth assays on *A. annua seedlings* using graphene concentrations of 0, 10, 20, 50, 100, and 200 mg/l. Seedlings exposed to graphene concentrations below 50 mg/l exhibited a better growth status, whereas more than 100 mg/l graphene was toxic to *A. annua* (Supplemental Figure 2). We therefore used graphene concentrations of 10 mg/l and 20 mg/l for detailed investigations of its beneficial effects on *A. annua*. After 2 months of cultivation, the plant heights doubled with 10 mg/l graphene and increased over 3-fold with 20 mg/l graphene compared with the control (Figure 1A and Supplemental Figure 3A). The fresh and dry weights of shoots increased slightly at 10 mg/l, and there was a significant increase at 20 mg/l (Supplemental Figure 3B and 3C). Seedlings exposed to graphene had longer root systems (Figure 1B), but root scanning and observations of root cross-sections revealed that total root surface area, root volume, and average root diameter were significantly reduced in these samples (Figure 1C and Supplemental Figure 3D–3G). However, because of the greater root system length, there were no significant differences in root fresh and dry weight

(Supplemental Figure 3H and 3I). Application of graphene at an appropriate concentration thus promoted the growth of *A. annua*.

## Graphene promoted biomass accumulation by enhancing nitrogen uptake and leaf photosynthesis

To evaluate the relationship between graphene and biomass accumulation, we performed RNA sequencing (RNA-seq) of leaf samples. Because of the dramatic growth differences among treatments, there were more than 10 000 differentially expressed genes (DEGs) for each comparison (Supplemental Figure 4). We performed Kyoto Encyclopedia of Genes and Genomes (KEGG) enrichment analyses on the upregulated DEGs. Enriched pathways included those related to primary metabolism (starch, fructose, etc.), secondary metabolism (terpenoid backbone biosynthesis, flavonoid biosynthesis, etc.) and nutrient assimilation (nitrogen metabolism) (Supplemental Figure 5).

Using clues from the RNA-seq data, we found that expression levels of genes related to nitrogen metabolism, transport, and signaling were induced by graphene (Figure 1F). To assess this, we concentrated first on nutrient absorption because root morphology had also been affected (Figure 1B and 1C). Root activity measured by the triphenyl tetrazolium chloride method was over 212 μg/h/g when seedlings were cultivated with 20 mg/l graphene and less than 200 μg/h/g with 10 mg/l graphene or in the absence of graphene (Supplemental Figure 3O), suggesting a dosage-dependent effect. Because nitrogen metabolism genes were upregulated, nitrogen assimilation was strengthened after graphene treatment, leading to greater nitrogen accumulation in the seedlings (Supplemental Figure 3M and 3N). We next used $^{15}NH_4Cl$ to feed hydroponic seedlings treated with graphene and detected the resulting isotope signals. Roots exposed to graphene absorbed more isotope-labeled ammonium: 115 μmol $^{15}N$/g root dry weight (DW) $h^{-1}$ in the 20 mg/l treatment and 92 μmol $^{15}N$/g root DW $h^{-1}$ in the 10 mg/l treatment, compared with 81 μmol $^{15}N$/g root DW $h^{-1}$ in the control treatment (Figure 1D). Leaves showed a similar trend (Figure 1E), indicating that more nitrogen was transported to the leaves after graphene treatment. These results demonstrated that graphene increased the ability of the seedlings to absorb, transport, and assimilate nitrogen.

The most highly enriched pathway was starch and sucrose metabolism (Supplemental Figure 5), suggesting that photosynthesis was affected by graphene. We therefore examined DEGs related to the photosynthetic system (Figure 1G) and found that genes encoding chlorophyll A-B binding protein, photosystem II (PSII) PsbR, and other photosynthetic proteins were upregulated. Measurements of photosynthetic pigments (Figure 1H) revealed that the total chlorophyll content was as high as 2.2 mg/g fresh weight (FW) under graphene treatment compared with 1.8 mg/g FW in the control treatment (Figure 1H). As a result, graphene treatment increased the maximal photochemical efficiency of PSII (Fv [variable fluorescence]/Fm [fluorescence maximum]) by ∼15% (Figure 1I and 1J). Consequently, the content of soluble sugars, the products of photosynthesis, was twice as high in seedlings exposed to 20 mg/l graphene as in the controls (Figure 1K). These results suggest that graphene promotes plant growth by enhancing photosynthesis and sugar accumulation.

## Changes in diversity of the rhizosphere bacterial community after graphene treatment

To further analyze the prospects for graphene application to *A. annua*, we dissected its mode of action from the rhizosphere soil to the aboveground plant parts. Because plants are sessile autotrophic organisms, their growth is dramatically affected by the soil environment; we therefore analyzed changes in the microbiome of the rhizosphere soil surrounding *A. annua*. We obtained a total of 1674 high-quality bacterial 16S rRNA sequences (Supplementary Table 1), and the Shannon–Wiener curve and species accumulation curve showed that the sequences were of high quality (Supplemental Figure 6A). Rarefaction curve analysis showed that 810–1064 bacterial operational taxonomic units OTUs (Supplemental Figure 6B) were clustered into 7248 OTUs (Supplemental Figure 6C). The bacterial OTUs were derived from 29 phyla, 527 genera, and 633 species (Supplementary Table 2). Bacterial diversity varied significantly ($p < 0.05$) after the 10 and 20 mg/l graphene treatments compared with the pre-treatment sample (Supplemental Figures 7 and 8).

The majority of the bacterial OTUs could be assigned to 10 major phyla (Supplemental Figure 9A), 8 of which (Proteobacteria, Bacteroidota, Acidobacteriota, Patescibacteria, Actinobacteriota, Myxococcota, Gemmatimonadota, and Bdellovibrionota) accounted for more than 90% of all bacterial OTUs (Supplemental Figure 9A). Among these 8 phyla, Acidobacteriota, Actinobacteria, Patescibacteria, Gemmatimonadota, and Bdellovibrionota increased dramatically in the 10 and 20 mg/l groups compared with the control group. However, levels of Bacteroidota were significantly lower ($p < 0.05$) in the treated groups than in the control group (Supplemental Figure 9A). At the family level of bacterial OTUs, differences were mainly caused by changes in the levels of Xanthobacteraceae, Micropepsaceae, Rhodanobacteraceae, and Caulobacteraceae (Supplemental Figure 9B). At the genus level of bacterial OTUs, levels of unclassified_Micropepsaceae, *Pseudolabrys*, *Dokdonella*, unclassified_LWQ8, and unclassified_Xanthobacteraceae were higher in the 10 and 20 mg/L groups compared with the control group, but levels of *Dongia* and *Flavobacterium* were lower (Supplemental Figure 9C). At the species level of bacterial OTUs, levels of unclassified_Micropepsaceae, unclassified_*Pseudo labrys*, unclassified_*Puia*, unclassified_LWQ8, and unclassified_Xanthobacteraceae were higher in graphene treatment groups compared with the control group, but levels of unclassified_*Dongia* and unclassified_*Flavobacterium* were lower (Supplemental Figure 9D).

## Co-occurrence network and differences in rhizosphere bacterial community diversity after graphene treatment

A co-occurrence network revealed that the bacterial community was dominated by six major genera: unclassified_Micropepsaceae, *Pseudolabrys*, unclassified_LWQ8, *Flavobacterium*, *Dokdonella*, and unclassified_*Xanthobacteraceae* (Figure 2A). The bacterial network was evenly divided between being positively and negatively correlated with an unequal number of bacteria in each category (Figure 2A). These results indicate that a core group of bacteria was present during *A. annua* growth.

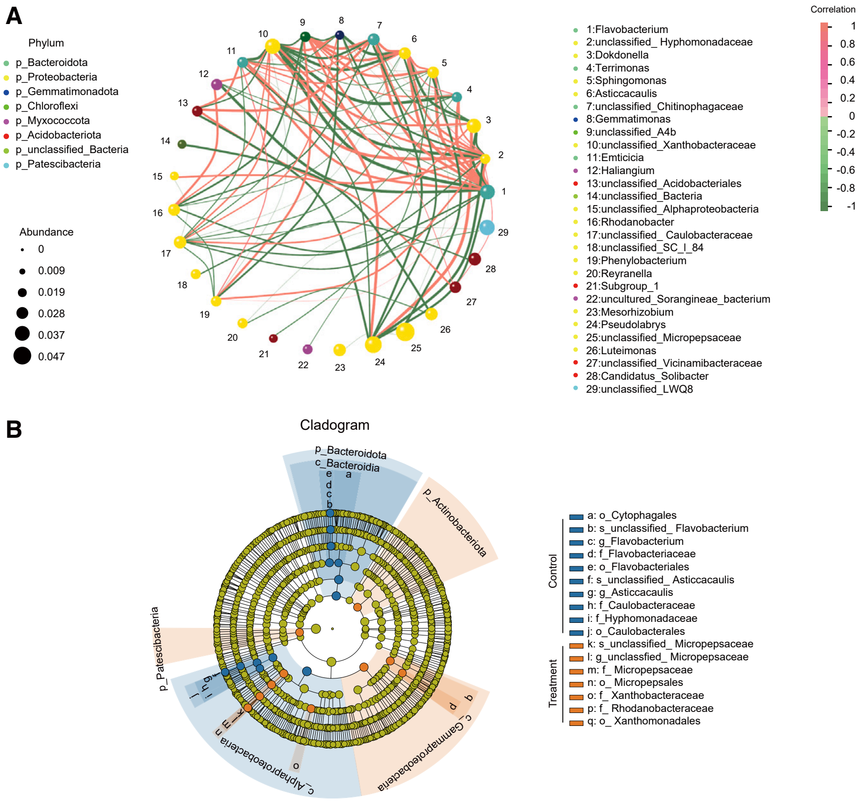


**Figure 2. Co-occurrence network and phylogenetic cladogram of bacterial LEfSe.**
**(A)** Co-occurrence network among the top 50 bacterial OTUs based on absolute abundance. Dot size indicates abundance, line thickness wrepresents correlation strength, dot color indicates the genus, orange lines indicate positive correlations, and green lines indicate negative correlations.
**(B)** Phylogenetic cladogram of bacterial LEfSe between the control and graphene treatment groups (10 mg/l and 20 mg/l). The phylum (p), class (c), order (o), family (f), and genus (g) names indicated by the letters are shown in the legend on the right. The circles from inside to outside represent classification levels from phylum to genus (or species). Each small circle at a given classification level represents a classification at that level, and the diameter of the circle corresponds to its relative abundance. Different colors indicate different groups, and nodes of different colors indicate groups of microorganisms that play an important role in the groups represented by the colors. Control group, samples cultivated without graphene; treatment group, combination of samples cultivated with 10 mg/l and 20 mg/l graphene.

As stated above, there were marked differences in richness of the rhizosphere microbial community after graphene treatment. A line discriminant analysis effect size (LEfSe) evolutionary branching diagram of bacteria between the control and graphene (10 and 20 mg/l) groups is shown in Figure 2B. At the bacterial taxonomic level, s-unclassified_Micropepsaceae, g-unclassified_Micropepsaceae, f-Micropepsaceae, o-Micropepsales, f-Xanthobacteraceae, f-Rhodanobacteraceae, and o-Xanthomonadales were significantly enriched in the 10 or 20 mg/l group (Figure 2B). Most of these taxa have been reported to promote plant growth and development by enhancing the carbon cycle and accelerating the decomposition of organic matter in rhizosphere soil (Kappler et al., 2012; Gutierrez, 2017).

## Graphene enters *A. annua* cells and maintains ROS homeostasis

To estimate the direct effects of graphene on *A. annua*, we used the Raman spectrum to detect the biodistribution of graphene. Raman signals indicated that graphene accumulated in the roots and leaves of treated samples (Figure 3B), whereas the signal was limited in control samples (Figure 3A).

Because graphene diffuses into cells, it could potentially cause injuries as foreign matter. To investigate the role of exogenous graphene in cell membrane damage, we first focused on the ROS scavenging system. Graphene concentrations of 10 and 20 mg/l significantly increased the concentration of $H_2O_2$ by 7.8% and 61.1%, respectively, compared with the control (Figure 3C). We next examined the activities of ROS-scavenging enzymes in detail. Superoxide dismutase (SOD) activity was significantly reduced by 23.8% and 29.8% in the 10 and 20 mg/l groups, respectively, compared with the control (Figure 3C). Interestingly, 10 mg/l graphene slightly increased peroxidase (POD) activity, whereas 20 mg/l graphene significantly reduced POD activity by 16.8% compared with the control (Figure 3C). Likewise, catalase (CAT) activity increased by 16.8% and 31.8% at graphene concentrations of 10 and 20 mg/l (Figure 3C). Compared with the control treatment, 20 mg/l graphene significantly reduced the activity of ascorbate peroxidase (APX) by 13.9% (Figure 3C); 10 mg/l graphene increased APX activity by approximately 2.50%, but this difference was not significant (Figure 3C).

Graphene has been reported to exhibit POD-mimicking activity that aids in ROS cleavage (Song et al., 2010). We therefore asked whether the graphene used in this study exhibited similar activity, and we used 0.1 mg of graphene to test for enzyme-mimicking reactions. Graphene alone exhibited no SOD (Figure 3D), CAT (Figure 3E), or POD (Figure 3F) enzyme-mimicking activity. This result could be attributable to differences in material characteristics, such as size or other properties, between our work and that reported previously. When we added 0.1 mg of SOD protein to the assay mixture together with different amounts of graphene, there was still no discernable effect of graphene on SOD activity (Figure 3D). However, when 0.1 mg of CAT or POD was combined with different concentrations of graphene, the CAT or POD enzyme activity increased in a dose-dependent manner (Figure 3E and 3F), indicating that graphene serves as a catalyst accelerator *in vitro*. In summary, even when limited graphene entered the cells at a low dosage, ROS levels increased. Plant cells could be protected by the dynamic ROS-scavenging system as well as by graphene's function as a catalyst enhancer *in vivo*.

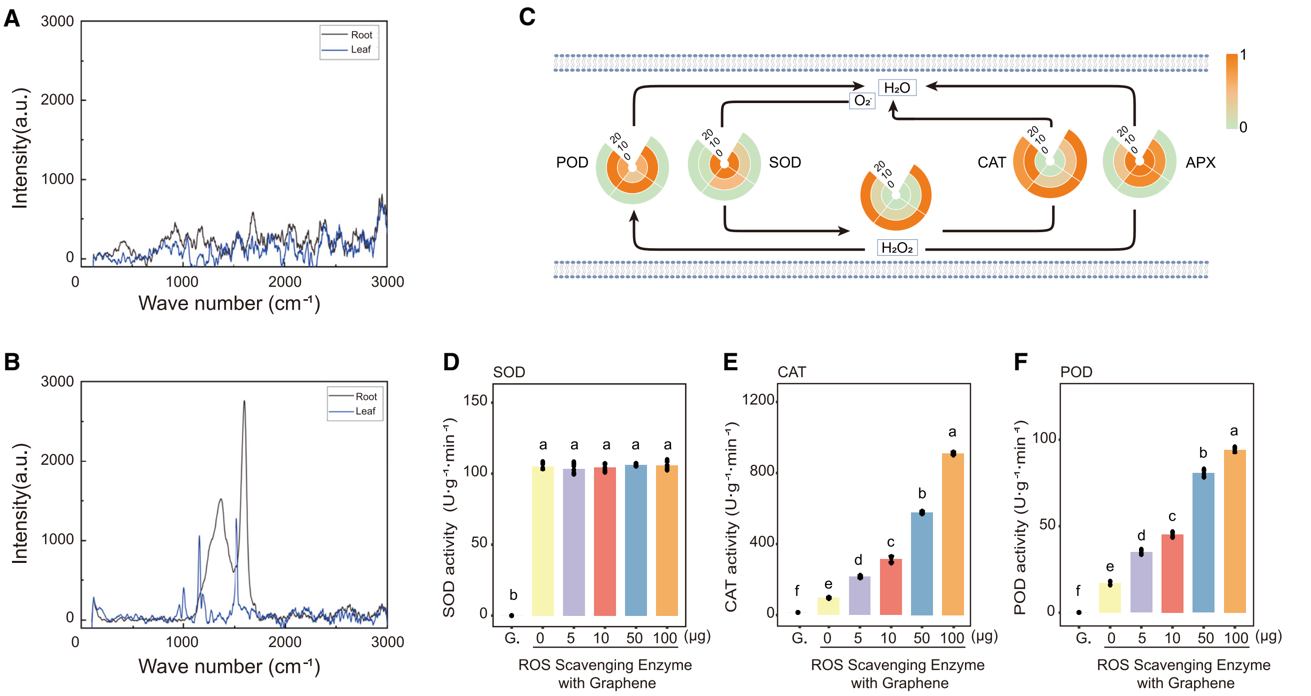


**Figure 3. Graphene induced ROS production and maintained ROS homeostasis by acting as a catalyst accelerator.**
**(A and B)** Graphene entered the seedlings. Raman spectra from roots and leaves without **(A)** or with **(B)** graphene treatment are shown.
**(C)** Heatmap of key antioxidant enzyme activities of the ROS-scavenging system affected by graphene. $H_2O_2$ content and activities of ROS-scavenging enzymes, including superoxide dismutase (SOD), peroxidase (POD), catalase (CAT), and ascorbate peroxidase (APX), are indicated by $\log_2$ and converted to a color scale after graphene treatment; triplicates were normalized between 0 and 1.
**(D–F)** ROS-scavenging enzyme activities of 0.1 mg graphene alone (G.) and 0.1 mg SOD **(D)**, CAT **(E)**, or POD **(F)** combined with 0, 5, 10, 50, or 100 μg graphene. Mean ± SD, n = 6. Different lowercase letters indicate a significant difference at $p < 0.05$ based on ANOVA.

## Graphene enhances environmental adaptation

Phytohormones are key regulators that enable plants to adapt to the environment. Previous studies have shown that JA and ABA have fundamental roles in *A. annua* growth, as well as GST initiation and artemisinin biosynthesis (Fu et al., 2021; Yuan et al., 2023; Zheng et al., 2023). KEGG analyses of the RNA-seq data showed that the plant hormone signal transduction pathway was enriched most (Supplemental Figure 10), and we therefore measured levels of endogenous hormones in the graphene-treated seedlings (Supplemental Figure 11). There were no differences in ABA content among treatments (Supplemental Figure 11A), but contents of other stress-related hormones increased in response to graphene application (Supplemental Figure 11B–11D). JA and its derivative Jasmonoyl-isoleucine (JA-ILE) were significantly upregulated because their biosynthetic pathway was enhanced (Supplemental Figure 11D), according to RNA-seq. Salicylic acid (SA) or salicylic acid 2-O-β-D-glucose (SAG) contents also trended upward (Supplemental Figure 11B and 11C) and benefitted from sufficient substrates supplied by dynamic phenylpropanoid biosynthesis (Supplemental Figure 10). The delivery of ROS-scavenging NMs has been reported to alleviate abiotic stress (Zhao et al., 2022). Applying graphene not only increases the activity of ROS-scavenging enzymes but also causes accumulation of stress-related phytohormones like JA and SA (Supplemental Figure 11), indicating that it has roles in both biotic and abiotic stress resilience. Interestingly, JA has been reported to be an essential phytohormone for artemisinin biosynthesis, revealing that graphene is a potential tool for the production of valuable natural products.

## Graphene promotes artemisinin accumulation in GSTs

Although graphene boosted the growth of *A. annua*, it remained unclear whether it could facilitate production of bioactive compounds. The biosynthesis of artemisinin, the most important natural product in *A. annua*, begins with the sesquiterpene FPP. The RNA-seq results suggested that expression levels of genes involved in terpenoid backbone biosynthesis were upregulated (Supplemental Figure 12), and we therefore measured the expression of genes encoding key enzymes of the specialized artemisinin pathway using quantitative real-time PCR. All genes of the biosynthetic pathway increased in expression after cultivation with graphene (Figure 4A) and were prospectively induced by the increased JA (Supplemental Figure 11D) (Ma et al., 2018). Accordingly, artemisinin and its substrate DHAA showed greater accumulation in the graphene-treated seedlings (Figure 4A).

GSTs are considered to be factories for valuable secondary metabolites. Because of the increased artemisinin levels in graphene-treated seedlings, we next examined the density of GSTs on leaves using SEM. GST density was about 30%–80% higher on leaves of seedlings treated with 10 mg/l and 20 mg/l graphene compared with controls (Figure 4B and 4C). We also examined the expression levels of genes encoding key transcription factors that regulate GST growth and found that such genes, including

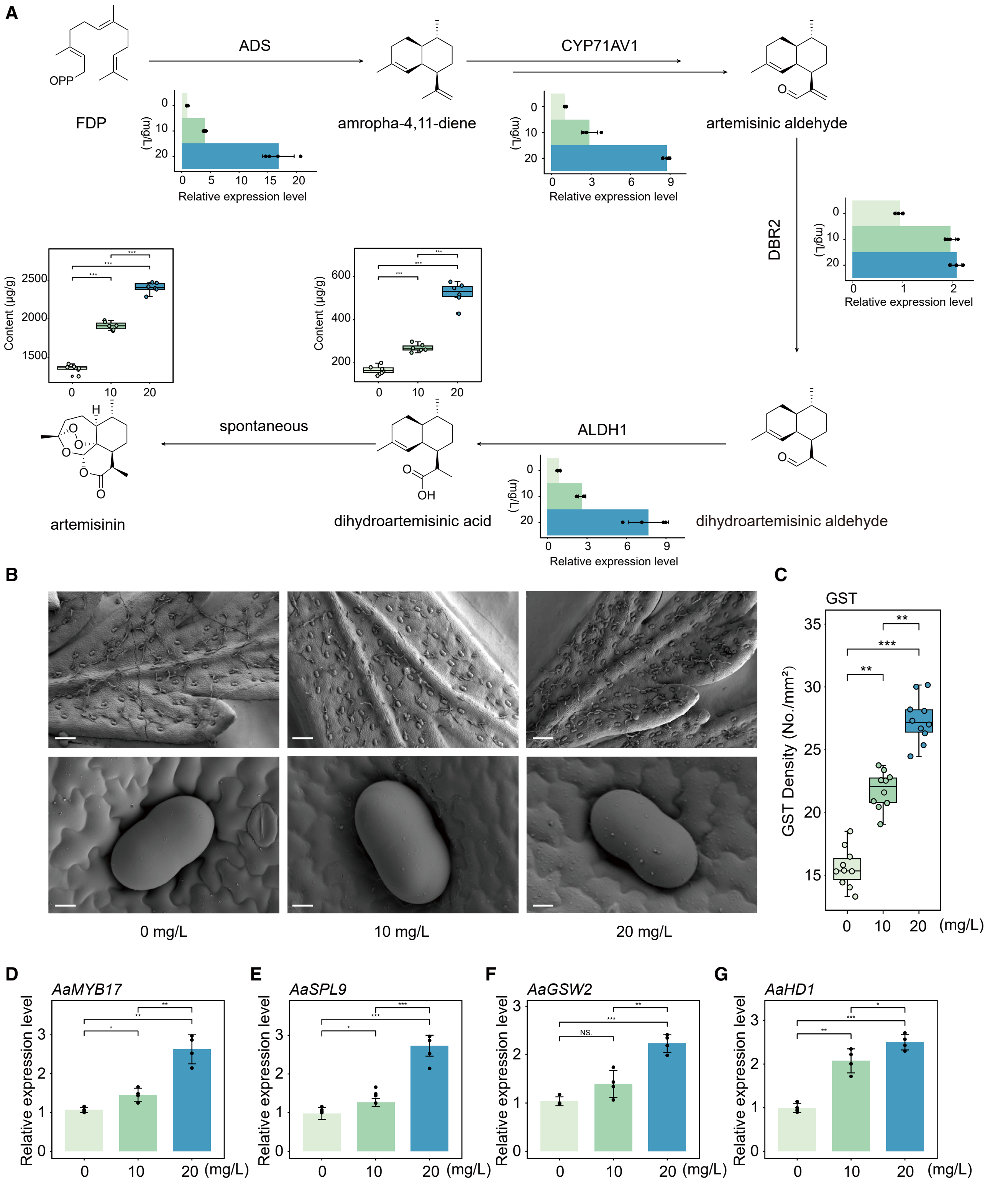


**Figure 4. Graphene promoted artemisinin biosynthesis and initiation of glandular secreting trichomes (GSTs).**

**(A)** Relative expression of genes encoding key enzymes in the specific pathway of artemisinin biosynthesis. Quantitative real-time PCR was performed using *AaActin* as the internal reference; expression in the 0 mg/l treatment was set to one (means of quadruplicates ± SD). The contents of dihydroartemisinic acid (DHAA) and artemisinin are shown above their chemical structure formulas (mean ± SD, n = 6, ***$p$<0.001, Student's $t$-test).

**(B)** Images of GSTs on leaves with and without graphene treatment. Scale bars: 200 μm (top row) and 10 μm (bottom row).

**(C)** GST density on leaves (mean ± SD, n = 10; **$p$<0.01, ***$p$<0.001, Student's $t$-test).

**(D–G)** Relative expression of genes involved in GST initiation (mean ± SD, n = 4; *$p$<0.05, **$p$<0.01, ***$p$<0.001, Student's $t$-test).

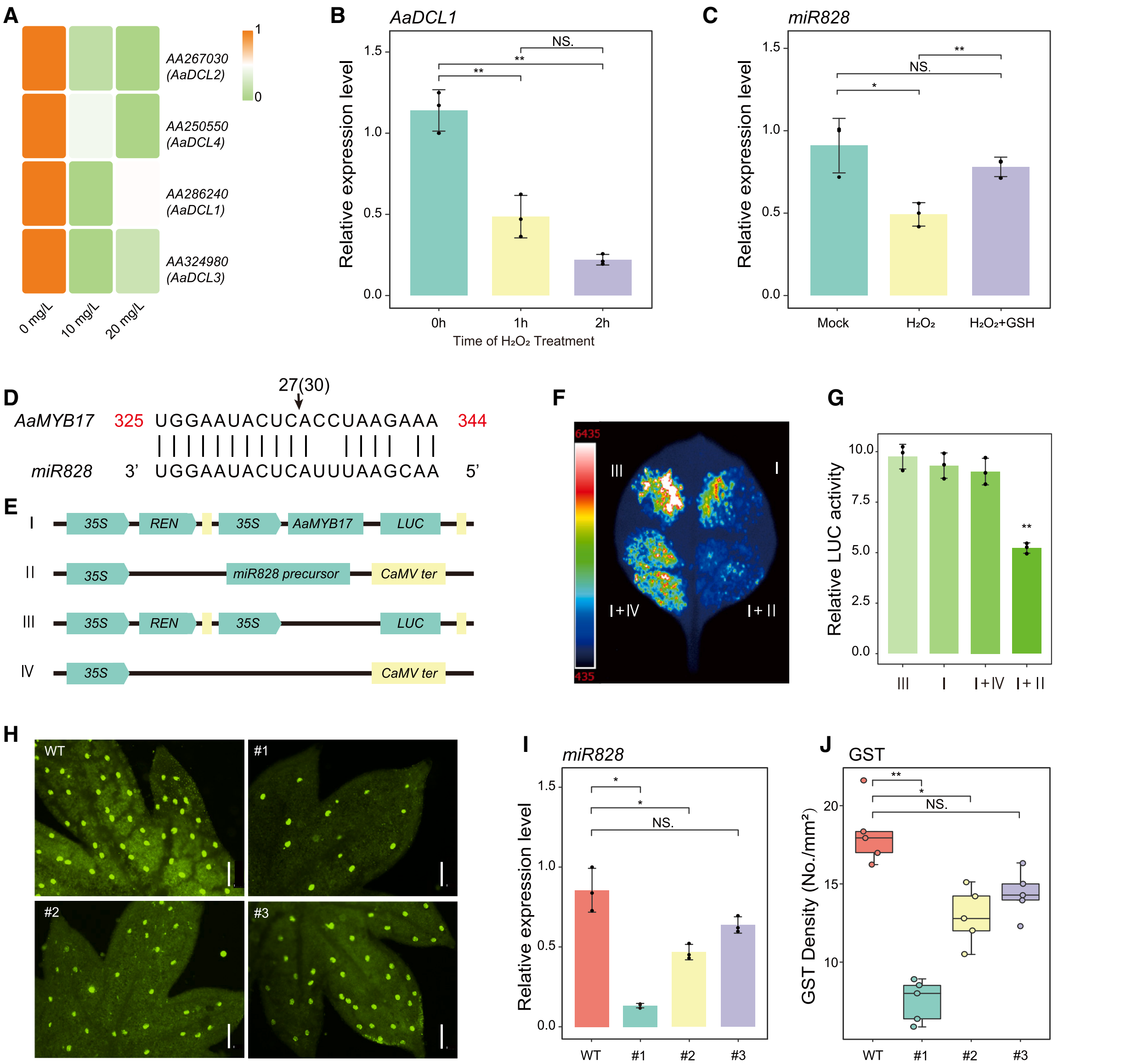


**Figure 5. Graphene blocked the biogenesis of *miR828* to increase GST density.**
**(A)** Heatmap of Dicers, which are responsible for small RNA biogenesis, generated from RNA-seq data.
**(B)** Relative expression of *AaDCL1* in *A. annua* treated with 1 mM $H_2O_2$. The level at 0 h (untreated) was set to 1 (means of triplicates ± SD).
**(C)** Relative expression of mature *miR828* after 1 mM $H_2O_2$ treatment or 1 mM $H_2O_2$ supplemented with 100 μM GSH. The level of the mock control was set to 1 (means of triplicates ± SD); U6 served as the internal reference. *$p$<0.05, **$p$<0.01, Student's $t$-test.
**(D)** Analysis for 5′ RACE. The red numbers indicate the gene positions, and the black arrow indicates the cleavage site; 27 of 30 reads were sequenced at this site.
**(E)** Schematic map of LUC reporters (I), effectors (II), and controls (III and IV).
**(F)** Image of LUC activities.
**(G)** Quantitative LUC activities. Mean ± SD, n = 3; **$p$<0.01, Student's $t$-test.
**(H–J)** Phenotypes of *miR828* overexpression lines.
**(H)** Image of GSTs on leaves from the wild type and three different overexpression lines. Scale bar: 200 μm.
**(I)** Relative expression of *miR828* in leaves as shown in **(H)** (mean ± SD, n = 3; *$p$<0.05, Student's $t$-test).
**(J)** GST densities of the samples from **(H)** (mean ± SD, n = 10; **$p$<0.01, Student's $t$-test).

*AaMYB17* (Figure 4D), *AaSPL9* (Figure 4E), and *AaGSW2* (Figure 4F), were significantly upregulated by graphene treatment. Expression of a downstream core gene for GST initiation, *AaHD1*, was also increased to activate this process (Figure 4G). These results demonstrated that graphene enhanced the initiation of GSTs and the biosynthesis of natural products.

## Graphene blocked the biogenesis of miRNAs to enhance GST initiation

miRNAs are key regulators of trichome initiation, and the RNA-seq data showed that expression of Dicer genes involved in miRNA biogenesis was reduced in the graphene-treated plants

(Figure 5A), suggesting that graphene might inhibit miRNA function. We therefore detected the expression of mature miRNAs in the seedlings. Expression of miRNAs reported to be responsible for NGTs or GSTs, including *miR156*, *miR160*, and *miR828* (Supplemental Figure 13), was significantly downregulated in graphene-treated plants. Expression of miR396, reported to be the most abundant miRNA in *A. annua* leaves (Khan et al., 2020), displayed a pattern similar to that of other miRNAs (Supplemental Figure 13A). This suggests that fewer miRNA precursors were spliced into their mature forms, possibly because of a low level of Dicers. Notably, a recent study in maize demonstrated that $H_2O_2$ could inhibit the expression of *miR169*, thereby enhancing salt tolerance (Xing et al., 2022). Our results indicate that exposure to a low concentration of graphene increased $H_2O_2$ content by approximately 60% despite activation of the ROS-scavenging system and promotion of enzyme activities by the NM (Figure 3). The observed ideal phenotypes (Figures 1 and 4) and the decrease in miRNAs (Supplemental Figure 13) suggest that increased $H_2O_2$ may act as a signaling molecule, as reported previously. To confirm this possibility, we treated the seedlings with 1 mM $H_2O_2$ and observed a downregulation of Dicer expression (Figure 5B and Supplemental Figure 14A–14C). Because of the substantial reduction in *miR828* levels (Supplemental Figure 13D), we chose to investigate this miRNA further. Expression of *miR828* was also reduced upon exposure to exogenous $H_2O_2$, and this reduction could be partially rescued by the ROS scavenger glutathione (GSH) (Figure 5C). In addition, we observed that *AaMYB17*, the putative target gene of *miR828*, exhibited an expression pattern opposite to that of this miRNA (Figure 4D). We performed 5′ rapid amplification of cDNA ends (RACE) to confirm *miR828* cleavage of *AaMYB17*, the key gene for GST initiation. The results showed that *miR828* bound to and cleaved *AaMYB17* at nucleotide 334 (Figure 5D). We then co-expressed the precursor of *miR828* as an effector alongside the *AaMYB17* coding sequence fused with the luciferase (LUC) reporter and driven by the cauliflower mosaic virus (CaMV) 35S promoter in *Nicotiana benthamiana* (Figure 5E). The LUC activity after co-expression was much lower than that observed after expression of AaMYB17-LUC alone (Figure 5F and 5G), implying that *miR828* could degrade *AaMYB17 in vivo*. To investigate whether *miR828* could regulate GSTs, we generated transgenic lines expressing its precursor. The results revealed that overexpression of *miR828* reduced trichome density by approximately 60% (Figure 5H–5J). Graphene could thus block the biogenesis of miRNAs and directly disrupt the function of the miR828–AaMYB17 module (Figure 5).

In addition to miRNAs, other small RNAs, such as small interfering RNAs (siRNAs), could also be affected by graphene because of Dicer downregulation (Supplemental Figure 14A–14C). To examine the effects of graphene on small RNAs, we applied graphene to the *AaMYB17* RNAi lines because the mode of action of RNAi is similar to that of miRNAs, i.e., siRNA is spliced by Dicers and forms the RNA-induced silencing complex (RISC) to degrade target genes. Consistent with a previous study (Qin et al., 2021), trichome numbers were markedly lower in the RNAi line compared with the control (Supplemental Figure 14D–14F). Application of 20 mg/l graphene to the RNAi lines significantly increased trichome numbers (Supplemental Figure 14D–14F). Similar to the results for *miR828*, the expression level of *AaMYB17* was higher in graphene-treated RNAi lines than in untreated lines (Supplemental Figure 14E), owing to the low efficiency of siRNA generation and function. Graphene-induced ROS could act on Dicer to remove the miRNAs/siRNAs that target critical genes in *A. annua* and enhance GST initiation.

## DISCUSSION

Non-point-source pollution has given rise to significant concerns about current agricultural production practices. Cost–benefit analysis has revealed that nanofertilizers and nanopesticides are valuable for increasing crop revenue and lowering environmental risk (Su et al., 2022; Lu et al., 2024). Here, we found that graphene could serve as a nanofertilizer to substitute for chemical fertilizer (Figure 6). Delivering ROS-scavenging NMs can alleviate abiotic stress (Zhao et al., 2022). Here, graphene application not only increased the activity of ROS-scavenging enzymes (Figure 3) but also caused accumulation of stress-related phytohormones like JA and SA (Supplemental Figure 11), indicating that it has roles in both biotic and abiotic stress resilience. Interestingly, JA has been reported to be an essential phytohormone for artemisinin biosynthesis, suggesting that graphene may be a reliable tool for production of valuable natural products. Although this nano-based strategy could reduce the release of chemicals, the release of small particles into the environment may still bring potential ecological risks. Thus, research has focused on the environmental impact of such particles, including micromaterials/NMs or NPs produced by agriculture. For example, microplastic residues on rice (Yu et al., 2022), wheat (Zhu et al., 2022), soybean (Lian et al., 2022) and cotton (Wu et al., 2022) fields could change the soil bacterial community structure and affect crop growth. Here, graphene increased beneficial microorganisms and promoted plant growth during cultivation of *A. annua* (Figure 2). Hence, introducing NMs to plant production could be an eco-friendly approach to reduce non-point-source pollution derived from agriculture.

*A. annua* is a traditional Chinese herb that produces the valuable compound artemisinin. Much effort has been dedicated to identification of key genes and molecular design breeding of this herb (Zheng et al., 2023), just as for cereals and other economically important crops. However, unlike field crops, *A. annua* germplasm resources usually have a highly heterozygous genetic background that limits both conventional and molecular breeding (Shen et al., 2018). In addition, although genetic transformation systems are well established for many crops, there is still a lack of satisfactory transgenic acceptors for *A. annua*, leading to low transgenic efficiency. Finally, public perception and strict policies restrict the commercialization of products from genetically modified organisms (Beumer, 2019). Therefore, improved crop cultivation practices deserve to be taken seriously. Here, physiological and bioinformatic data showed that nutrient absorption and photosynthesis were enhanced by graphene (Figure 1), resulting in greater *A. annua* biomass (Figure 1). Notably, major pharmaceutical components also showed greater accumulation in GSTs (Figure 4), suggesting that this graphene-based cultivation strategy represents a new approach to solving the problem of low artemisinin content. The successful application of NMs could demonstrate the ability of crop cultivation strategies to overcome the limitations of molecular breeding.

*A. annua* is a perfect model plant for research on GSTs and secondary metabolites. In basic research, more studies have concentrated

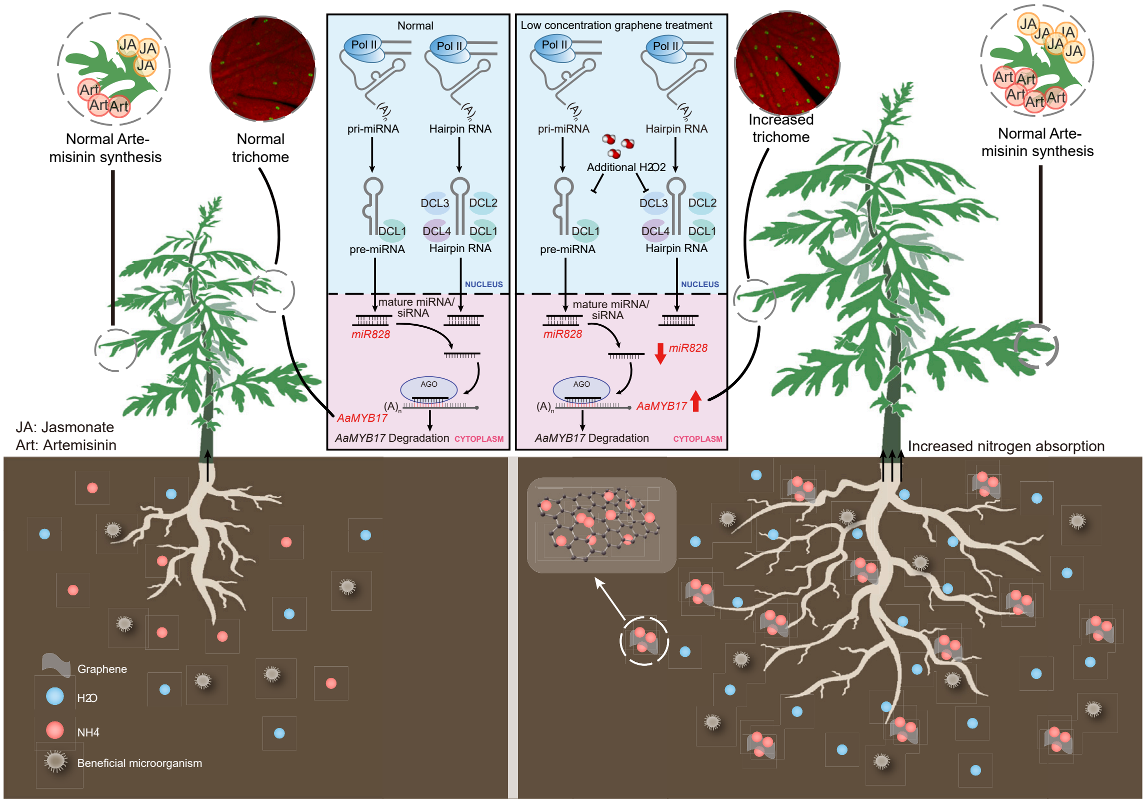


**Figure 6. Proposed model showing how graphene serves as a nanofertilizer to promote plant growth and artemisinin production.**
Introduction of graphene into *A. annua* cultivation has comprehensive effects on plants. In the context of the environment, a low dose of graphene alters bacterial community structure, enhancing the carbon cycle. In terms of physiological effects, graphene enhances environmental adaptation by increasing the content of phytohormones such as JA. Simultaneously, improved root activity facilitates greater nitrogen absorption and translocation to leaves in conjunction with graphene sheets, resulting in enhanced photosynthesis and increased biomass accumulation. At the molecular level, miRNA biogenesis is inhibited by an appropriate level of $H_2O_2$. For example, the miR828–AaMYB17 module is regulated by graphene, contributing to GST initiation. Induction of ROS, JA, and miRNA-targeted genes that positively regulate GST initiation increases the synthesis of artemisinin, a valuable natural product. Application of graphene is thus a potential strategy for cultivation of traditional Chinese medicinal plants.

on the nonglandular single-cell trichomes of plants like *Arabidopsis*. The R2R3 MYB, basic-helix-loop-helix, and WD 40 transcription factors, which form the GL1–GL3/EGL3–TTG1 complex to activate the downstream HD-ZIP IV gene *GL2*, comprise the basic model of trichome initiation (Cui et al., 2022). Although this model is conserved, to some extent, in different plants such as cotton (Wang et al., 2019), there are still several differences between GSTs and NGTs. Because of the scientific and economic value of GSTs in *A. annua*, more attention should be paid to the mechanisms and engineering of their traits. Our assays showed that graphene reduced the biogenesis of miRNAs/siRNAs, leading to increased expression of their target genes and thus to increased GST density (Figure 5 and Supplemental Figure 14). A recent study showed that graphene oxide NPs loaded with siRNAs could be taken up by plant cells, leading to gene silencing in intact cells (Li et al., 2022). On this basis, although graphene would neutralize the function of miRNAs through Dicers, we could still overexpress specific miRNAs/siRNAs delivered by graphene to explore their functions. CRIPSR is a powerful tool for the creation of desired traits, but it also suffers from the public perceptions and technical concerns discussed above (Mitter and Hussey, 2019). With the assistance of NMs like graphene, single-guide RNAs (sgRNAs) could be delivered and diffused into particular cells to produce non-transgenic, genome-edited plants, overcoming limitations to genome editing in various species and genotypes (Landry and Mitter, 2019). For example, genetic engineering could be performed in the GSTs alone through microinjection of nanocargoes to enhance artemisinin yield in the future. Because we harvest natural products from the GSTs and this strategy is much closer to precision cultivation rather than breeding, application of NMs to *A. annua* might help to quell public fears and maintain compliance with policies.

Finally, the graphene used in this study was produced in-house through an electrochemical method, resulting in a cost-effective material suitable for large-scale agricultural applications. Using this production process, we obtained a graphene solution at a cost of less than 3 Chinese Yuan (CNY)/l. With a solid graphene content of 5.0 g/l, the cost was further reduced to 0.6 CNY/g. In field production, approximately 3000 *A. annua* plants are cultivated per mu, with 0.1 g of graphene applied to each plant, thus requiring 300 g of graphene per mu for 180 CYN/mu. Farmers typically harvest around 200 kg of dry leaves per mu, resulting in an income of 1000–2000 CNY per mu (Nadali et al., 2014; Kung et al., 2018). Our experiments demonstrated that graphene exposure increased dry leaf weight by 20% and increased artemisinin

content by approximately 5% per unit weight in the treated dry leaves (Supplemental Figure 15). This increase could potentially lead to incomes of 210–420 CYN/mu. Considering the reduced need for fertilizers and the promoted life cycle, revenue could be even higher.

The use of graphene in crops may cause potential environmental health and safety issues for consumers. Researchers investigated the uptake, transformation, distribution, and elimination of $^{14}C$-labeled graphene in rice (Huang et al., 2018) and found that $^{14}C$-labeled graphene could react with OH in leaves, leading to degradation of graphene into $^{14}CO_2$. After 15 days, the accumulation of graphene in stems and leaves disappeared, and no graphene was detected in rice seeds (Huang et al., 2018). In addition, because graphene has a polycyclic structure similar to that of lignin and polycyclic aromatic hydrocarbons, it can be degraded by lignin peroxidase enzymes secreted by microorganisms in the soil environment (Lalwani et al., 2014). Some soil bacteria can utilize graphene as a carbon source to support their growth (Qu et al., 2018). Here, we observed that graphene diffused into plant cells and was transferred to the leaves (Figure 3A and 3B). However, the Raman signal was relatively weak in leaves 1 month after exposure (Figure 3A and 3B), and it may disappear when the leaves are harvested, in line with findings in rice (Huang et al., 2018). Moreover, any remaining graphene could also be removed during the extraction process. These studies can help alleviate public safety concerns regarding the use of engineered graphene in agricultural crop production.

## METHODS

### Graphene preparation and characterization

The graphene used in this study was prepared in-house by an electrochemical method (Chen et al., 2022b). In brief, graphite was used as both the anode and cathode with distilled water as the electrolyte. The graphite electrode was electrolyzed and oxidized by a high-frequency pulse current to prepare graphene oxide. Through the action of an electrochemical electric field, external electrolyte ions (molecules) were inserted into the layered materials, like liquid phase stripping, while an electric field force was applied to drive electrolyte molecules to intercalate into the graphite cathode directly in an electrochemical manner. Thus, the graphite layer spacing became larger, and the van der Waals forces between the layers became weaker. Graphene was thus prepared by electrochemical stripping of graphite using a nonoxidizing method. The characteristics of graphene were analyzed by UV-visible and Raman spectroscopy (Horiba, LabRAM HR Evolution). Raman spectra were obtained using a Renishaw inVia Qontor with a 532-nm excitation laser. Graphene morphology was examined by SEM (Tescan MAIA3 LMH) and transmission electron microscopy (TEM; Tecnai G2 F20 S-TWIN TMP).

### Rhizosphere soil sampling, soil DNA extraction, and Illumina HiSeq 2500 sequencing

Rhizosphere soil samples were collected from the 0 mg/l, 10 mg/l, and 20 mg/l groups. There were six biological replicates per treatment, each obtained by mixing five random rhizosphere soil samples. Samples were frozen with liquid nitrogen and stored in a freezer at −80°C prior to extraction of soil DNA for amplicon sequencing. Total DNA was extracted from each soil sample (0.3 g) using the NucleoSpin 96 Soil kit (Macherey-Nagel, Germany); 30 ng of soil DNA was used for subsequent PCR analysis. Primers 338F (5′-ACTCCTACGGGAGGCAGCAG-3′) and 806R (5′-GGACTACHVGGGTWT CTAAT-3′) were used to amplify the V3–V4 region of the prokaryotic 16S rRNA gene. The PCR products were checked using 1% agarose gel electrophoresis and recovered using the Agarose Gel Extraction Kit (GeneJET, Thermo Scientific, USA). Amplicon library preparation and 150-bp paired-end DNA sequencing on the Illumina HiSeq 2500 platform were performed at Beijing Biomarker Technologies (Beijing, China).

### Species annotation and taxonomy analysis

Clean tags with at least 97% similarity were clustered into OTUs using USEARCH v.10.0 (Edgar, 2013) and filtered using the 0.005% OTU abundance filtering approach (Bokulich et al., 2013). The SILVA database (v.138; http://www.arb-silva.de) and the UNITE database (v.7.2; https://unite.ut.ee) were used to identify the bacterial OTU-representative sequences, and the RDP classifier algorithm was used with a 0.8 confidence threshold (Wang et al., 2007; Abarenkov et al., 2010; Quast et al., 2013).

High-quality OTU sequences from the bacterial group were aligned to the microbial reference database (release 132 [http://www.arb-silva.de] and release 8.0 [https://unite.ut.ee/]) to annotate each OTU with the corresponding species classification information, including phylum, class, order, family, genus, and species. QIIME 2 software was then used to generate a species-level abundance table (Caporaso et al., 2010b; Lawley and Tannock, 2017; Estaki et al., 2020), and community structures were drawn at the taxonomic level using R (v.4.0.2; https://www.r-project.org). The bacterial sequences were aligned and a neighbor-joining phylogenetic tree constructed using PyNAST software (v.1.2.2; http://biocore.github.io/pynast/; Caporaso et al., 2010a).

### Diversity analysis

The alpha diversity indices (Chao1 index, Ace index, Shannon index, and Simpson index) of the samples were evaluated using Mothur v.1.30 (Grice et al., 2009). A beta diversity analysis was performed using QIIME 2 (Jiang and Takacs-Vesbach, 2017; Jiang et al., 2022). Dataset normalization for the alpha and beta diversity analyses was completed using a rarefaction curve analysis to equal depth. Principal-component analysis, analysis of similarities (ANOSIM), and LEfSe (Segata et al., 2011) were performed using R (v.4.0.2), and SPSS (v.19.0) was used to perform the significance analysis. The LefSe analysis was used to screen for biomarkers and compare q values to determine the significance of differences between the three groups at each classification level (Segata et al., 2011).

### Plant materials and graphene exposure

The sequenced cultivar Huhao 1 was used as the standard genotype for the assays. Huhao 1 seedlings and AaMYB17-RNAi transgenic lines were cultivated in a greenhouse at a controlled temperature of 25°C ± 2°C with a 16-h light/8-h dark photoperiod. Different concentrations of graphene (0, 10, 20, 50, 100, and 200 mg/l) were applied to wild-type or AaMYB17-RNAi transgenic seedlings when the first two true leaves had unfolded and the plants had reached the 2-week stage. Plants were grown in pots with 200 g of soil, and 100 mL of graphene solution was added to each pot every 3 days for 2 weeks, with water serving as the control. Plant height measurements were taken following the treatments. *N. benthamiana*, used for transient transformation, was grown under the same conditions as *A. annua*.

The miRNA precursor was synthesized by GenScript (Nanjing, China) and ligated into the vector to construct pHB-miR828. The overexpression construct was then introduced into *Agrobacterium tumefaciens* strain EHA105 for subsequent transformation into *A. annua* as described previously (Ma et al., 2018). Phenotypic changes in *A. annua* plants, including those transformed with the empty vector (control plants) and *miR828* overexpression lines, were monitored at specified intervals under the standard growth conditions described above.

### Statistical analysis

Statistical analysis was performed using one-way ANOVA followed by Student's *t*-tests in R v.4.3.1 with the ggplot package. More than 50

seedlings with uniform characteristics were selected for the control group and each treatment group. Unless otherwise specified, ten seedlings were randomly selected for tests and subsequent statistical analyses.

### Measurement of plant weight and root morphology

Two-month-old washed seedlings were dissected into shoots and roots for fresh weight measurement. The fresh roots were then scanned using a dual-lens scanning system (V700, Seiko Epson, Japan) as described previously (Chen et al., 2022b).

For root morphology, the basal part of the treated or untreated roots was fixed in formaldehyde–acetic acid solution, dehydrated in a graded ethanol series, and embedded in Paraplast. A rotary microtome (Leica RM2235) was used to section the samples to a 10-μm thickness. The sections were stained with toluidine blue and observed under a light microscope (BX51, Olympus, Tokyo, Japan).

### ROS and antioxidant enzyme measurements

The activities of antioxidant enzymes were measured according to a published method (Tian et al., 2019). In brief, 0.5 g of frozen leaf sample was sliced and homogenized in an ice-cold pestle, 5 ml of extraction buffer (50 mM [pH 7.8]) containing 0.2 mM EDTA and 0.4% polyvinylpyrrolidone (w/v) was added, and the mixture was centrifuged at 10 000 *g* and 4°C for 20 min. The supernatant was collected for subsequent assays. Total protein content was determined beforehand using the Coomassie brilliant blue reaction at 595 nm. SOD activity was assessed by measuring the ability of the extract to inhibit the photochemical reduction of nitroblue tetrazolium (NBT) at 560 nm. POD activity was measured using guaiacol as a substrate at 470 nm. CAT activity was measured as a decline in absorbance at 240 nm due to oxidation of $H_2O_2$. To measure $H_2O_2$ concentration, absorbance of the titanium peroxide complex was detected at 410 nm. Spectrophotometric measurements were performed with a UV-visible spectrophotometer (UV3200, Mapada Instruments, China).

For $H_2O_2$ treatment, 2-week-old seedlings were treated with 1 mM $H_2O_2$ or 1 mM $H_2O_2$ supplemented with 100 μM GSH. Leaves were harvested 0, 1, and 2 h after treatment for gene expression analysis.

To assess enzyme-mimicking activities, solutions containing 0, 5, 10, 50, and 100 μg graphene were tested alone. To assess the ability of graphene to promote enzyme activities, 0.1 mg SOD, CAT, or POD (Sinopharm Chemical Reagent) was combined with 0, 5, 10, 50, and 100 μg graphene, and enzyme activities were measured using the same methods.

### Chlorophyll fluorescence, total chlorophyll content, and soluble sugar content

For chlorophyll fluorescence measurements, images of seedlings were captured with a pulse amplitude-modulated fluorimeter (IMAG-MAXI, Heinz Walz, Effeltrich, Germany) after a 30-min dark adaptation period, and the fluorescence signals were recorded. Fv/Fm images were also exported, and a representative leaf from each treatment is shown in Figure 1.

For measurement of total chlorophyll content, pigments were extracted from 0.1 g of fresh leaf tissue using 25 ml anhydrous ethanol and acetone (1:1 [v/v]) solution. The mixture was incubated in the dark for 12 h, and chlorophyll content was detected colorimetrically at 647 and 663 nm.

For measurement of soluble sugar content, 0.1 g dry leaf powder was extracted in 8 ml 80% (v/v) ethanol at 80°C and centrifuged; the supernatant was retained, and the pellet was re-extracted twice. The combined ethanol extracts were used for measurement of total soluble sugar content at 620 nm with the anthrone method (Tian et al., 2019).

### Nitrate uptake, total nitrogen content, and root system activity measurements

Seedlings cultivated in soil with or without graphene were transferred to a nitrogen-free hydroponic medium for 24 h. They were then washed with 0.1 mM $CaSO_4$ for 1 min and incubated for 30 min in hydroponic liquid containing 5 mM $^{15}NH_4Cl$ with a 99% atom excess of $^{15}N$. After labeling, seedlings were transferred to 0.1 mM $CaSO_4$ for 1 min. Ultrapure water was used to wash the separated shoots and roots at least four times. The $^{15}N$ content was determined with a continuous-flow isotope ratio mass spectrometer (DELTA V Advantage + Flash 2000, Thermo Scientific). Micro-Kjeldahl analysis was used to measure total nitrogen content (Xie et al., 2022). Root system activity of fresh root samples (0.03 g) was evaluated using the triphenyl tetrazolium chloride method (Gillissen and Becher, 1957).

### Nucleic acid isolation and expression analysis

Total RNA was extracted from *A. annua* with the RNAprep Pure Plant Kit (DP441, Tiangen Biotech, Beijing, China). Total RNA (1 μg) was used for cDNA synthesis with TransScript II First-Strand cDNA Synthesis SuperMix (AH301-03, TransGen Biotech, Beijing, China). The 10-fold diluted products were used for quantitative real-time PCR using SYBR Green *Pro* Taq HS Premix (AG11701, Accurate Biotechnology (Hunan), Changsha, China). miRNAs were isolated with the miRcute Plant miRNA Isolation Kit (Tiangen Biotech) according to the manufacturer's protocol. Reverse transcription was performed with the miRcute Plus miRNA First-Strand cDNA Kit, and the miRcute Plus miRNA qPCR Kit (Tiangen Biotech) was used for quantitative real-time PCR with the primers listed in Supplementary Table 3.

RNA-seq was performed by Biomarker Technologies using the ninth true leaves, counted from the top of the seedlings. Clean data were obtained by eliminating reads that contained adapters, poly-N sequences, and low-quality reads from the raw data. Clean data also underwent calculations to determine the Q20, Q30, and GC content and the level of sequence duplication. All subsequent analyses relied on the use of clean, high-quality data. Genes were annotated by searching against the published *A. annua* genome data (Shen et al., 2018) and categorized using the KEGG pathway database. The gene expression was first normalized and quantified as reads per kilobase per million reads. DEGs, identified using a false discovery rate of $\geq$0.001 and an absolute $\log_2$ ratio of $\leq$1, were determined using a statistical random test ($p < 0.05$).

5′ RACE was performed according to the instructions of the SMARTer RACE 50/30 Kit (Clontech Laboratories). In brief, a 5′-terminal adapter was added to total RNA (1 mg) extracted from tender leaves for reverse transcription. The *AaMYB17*-specific primer was used for 5′ RACE PCR, and the amplified product was fused to a cloning vector for sequencing analysis.

The dual-LUC system (Zhao et al., 2018) was modified for miRNA-cleaved target genes. The CaMV 35S promoter was amplified to drive the target gene, *AaMYB17*, ligated to the firefly LUC reporter gene. The *miR828* precursor was inserted into an expression vector. The vectors were transferred into *A. tumefaciens* harboring the co-suppression repressor plasmid pSoup-P19. The transformed cells were mixed in pairs, as shown in Figure 5, and infiltrated into *N. benthamiana* leaves. After 3 days of cultivation, leaf fluorescence was captured, and treated areas were harvested to detect the fluorescence values of LUC and *Renilla* LUC using the Dual-Luciferase Reporter Assay System Kit (E1910, Promega) with a luminometer (GloMax 20/20, Promega).

### Microscopy observation

Observation of GSTs was performed as described previously (Qin et al., 2021). In brief, a fluorescence microscope (Olympus) was used to scan the top, middle, and bottom of each leaf under excitation at 450–480 nm. Trichomes were counted using ImageJ software (http://rsb.info.nih.gov/ij).

For surface structures, samples were dried to a critical point in a dryer (Balzers CPD 020), then affixed to a brass disc with double-sided adhesive silver tape. A sputter-coating unit (Balzers CSD 004) was used to coat the samples with gold/palladium. After preparation, the samples were observed under a scanning electron microscope (Amray 1830 I). For TEM, a Leica EM UC6 ultramicrotome was used to make ultrathin sections, which were scoped at 80 kV with a Philips CM 12 transmission electron microscope.

### Artemisinin content measurement

The ninth leaf was collected from seedlings for measurement of artemisinin biosynthesis, and whole leaves were collected from 4-month-old plants for calculation of artemisinin yield. To obtain artemisinin, 0.1 g of dried leaves was extracted with methanol according to a previously reported method (Lu et al., 2013; Chen et al., 2021). In brief, dried leaf powder was subjected to two rounds of extraction with 2 ml of methanol using a model JYD-650 ultrasonic processor (Shanghai Zhisun Instrument, Shanghai, China). The mixture was then centrifuged for 10 min to remove suspended particles, and the final supernatant was filtered through a 0.25-μm filter.

Samples were analyzed using an Alliance 2695 high performance liquid chromatography (HPLC) system equipped with a 2420 ELSD detector (Waters, Milford, MA, USA). For artemisinin analysis, the HPLC parameters included a Waters C18 column (YMC-Pack ODS-A; particle size, 5 μm; pore size, 12 nm; column dimensions, 4.6 × 250 $mm^2$), a mobile phase of water–methanol (40:60 [v/v]), and a flow rate of 1 ml/min. The ELSD settings were optimized with a nebulizer gas pressure of 345 kPa, a drift tube temperature of 45°C, and a gain setting of 7 min.

DHAA was analyzed using the following HPLC conditions: a Waters C18 column (YMC-Pack ODS-A; particle size, 5 μm; pore size, 12 nm; column dimensions, 4.6 × 250 $mm^2$); mobile phase, acetonitrile–0.1% aqueous acetic acid (pH 3.2, 60:40 [v/v]); flow rate, 1 mL/min. ELSD parameters were optimized with a nebulizer gas pressure of 345 kPa, drift tube temperature of 45°C, and a gain setting of 13 min. Artemisinin from Sigma and DHAA from Guangzhou Honsea Sunshine Bio Science and Technology (Guangzhou, Guangdong, China) were used as reference standards and for construction of standard curves. Each sample was injected into a volume of 20 μl, and the results were processed and analyzed using Empower, the Waters chromatography data software.

## DATA AND CODE AVAILABILITY

All data supporting the findings of this study are available within the article and its supplemental information files and are available from the corresponding author upon request. Raw sequencing data have been deposited in the GSA (accession number: CRA010136) and are publicly accessible at https://ngdc.cncb.ac.cn/gsa.

## SUPPLEMENTAL INFORMATION

Supplemental information is available at *Plant Communications Online*.


## FUNDING

This work was supported by the National Natural Science Foundation of China (82274047 and 52071192), the National Key R&D Program of China (2018YFA0900600), the Engineering Research Center of Coal-Based Ecological Carbon Sequestration Technology of the Ministry of Education (MJST2023-3), the China Postdoctoral Science Foundation (2023M732232), and SJTU Trans-med Awards Research (20190104). This work was also supported in part by the Bill & Melinda Gates Foundation (OPP1199872 and INV-027291). Under the grant conditions of the Foundation, a Creative Commons Attribution 4.0 Generic License has already been assigned to the Author Accepted Manuscript version that might arise from this submission.


## AUTHOR CONTRIBUTIONS

J.C., T.S., Z.C., and K.T. conceived the research. J.C., N.Y., L.W., J.L., L.H., T.W., J.W., and C.H. performed the experiments. J.C., L.W., T.S., W.Q., Y.W., Z.L., S.u.R., J.Q., J.Z., Y.S., and C.L. contributed materials and/or analyzed data. J.C., T.S., Z.C., and K.T. wrote the article.


## ACKNOWLEDGMENTS

We thank the Instrumental Analysis Center of Shanghai Jiao Tong University for the metabolite analysis. We thank X.-Y. Gao, J.-Q. Li, and Z.-P. Zhang from the CAS Center for Excellence in Molecular Plant Sciences for assistance with SEM. No conflict of interest is declared.

Received: June 15, 2023
Revised: October 9, 2023
Accepted: October 30, 2023
Published: November 2, 2023


## REFERENCES


**Abarenkov, K., Nilsson, R.H., Larsson, K.H., Alexander, I.J., Eberhardt, U., Erland, S., Hoiland, K., Kjoller, R., Larsson, E., Pennanen, T., et al.** (2010). The UNITE database for molecular identification of fungi - recent updates and future perspectives. New Phytol. **186**:281–285.

**Avouris, P.** (2010). Graphene: electronic and photonic properties and devices. Nano Lett. **10**:4285–4294.

**Beumer, K.** (2019). On the elusive nature of the public. Nat. Nanotechnol. **14**:510–512.

**Bokulich, N.A., Subramanian, S., Faith, J.J., Gevers, D., Gordon, J.I., Knight, R., Mills, D.A., and Caporaso, J.G.** (2013). Quality-filtering vastly improves diversity estimates from Illumina amplicon sequencing. Nat. Methods **10**:57–59.

**Borgatta, J., Ma, C., Hudson-Smith, N., Elmer, W., Plaza Pérez, C.D., De La Torre-Roche, R., Zuverza-Mena, N., Haynes, C.L., White, J.C., and Hamers, R.J.** (2018). Copper Based Nanomaterials Suppress Root Fungal Disease in Watermelon (Citrullus lanatus): Role of Particle Morphology, Composition and Dissolution Behavior. ACS Sustain. Chem. Eng. **6**:14847–14856.

**Cao, J.F., Zhao, B., Huang, C.C., Chen, Z.W., Zhao, T., Liu, H.R., Hu, G.J., Shangguan, X.X., Shan, C.M., Wang, L.J., et al.** (2020). The miR319-Targeted GhTCP4 Promotes the Transition from Cell Elongation to Wall Thickening in Cotton Fiber. Mol. Plant **13**:1063–1077.

**Caporaso, J.G., Bittinger, K., Bushman, F.D., DeSantis, T.Z., Andersen, G.L., and Knight, R.** (2010a). PyNAST: a flexible tool for aligning sequences to a template alignment. Bioinformatics **26**:266–267.

**Caporaso, J.G., Kuczynski, J., Stombaugh, J., Bittinger, K., Bushman, F.D., Costello, E.K., Fierer, N., Peña, A.G., Goodrich, J.K., Gordon, J.I., et al.** (2010b). QIIME allows analysis of high-throughput community sequencing data. Nat. Methods **7**:335–336.

**Chae, S., Le, T.H., Park, C.S., Choi, Y., Kim, S., Lee, U., Heo, E., Lee, H., Kim, Y.A., Kwon, O.S., et al.** (2020). Anomalous restoration of sp(2) hybridization in graphene functionalization. Nanoscale **12**:13351–13359.

**Chalvin, C., Drevensek, S., Dron, M., Bendahmane, A., and Boualem, A.** (2020). Genetic Control of Glandular Trichome Development. Trends Plant Sci. **25**:477–487.

**Chen, T., Li, Y., Xie, L., Hao, X., Liu, H., Qin, W., Wang, C., Yan, X., Wu-Zhang, K., Yao, X., et al.** (2021). AaWRKY17, a positive regulator of artemisinin biosynthesis, is involved in resistance to Pseudomonas syringae in Artemisia annua. Hortic. Res. **8**:217.

**Chen, Z., Zhao, J., Cao, J., Zhao, Y., Huang, J., Zheng, Z., Li, W., Jiang, S., Qiao, J., Xing, B., et al.** (2022a). Opportunities for graphene, single-walled and multi-walled carbon nanotube applications in agriculture: A review. Crop Design **1**, 100006.

**Chen, Z., Zhao, J., Qiao, J., Li, W., Guan, Z., Liu, Z., Bai, X., Xing, B., Zhang, J., Li, J., et al.** (2022b). Graphene-mediated antioxidant enzyme activity and respiration in plant roots. ACS Agric. Sci. Technol. **2**:646–660.

**Cui, W., Chen, Z., Shangguan, X., Li, T., Wang, L., Xue, X., and Cao, J.** (2022). TRY intron2 determined its expression in inflorescence activated by SPL9 and MADS-box genes in Arabidopsis. Plant Sci. **321**, 111311.

**Edgar, R.C.** (2013). UPARSE: highly accurate OTU sequences from microbial amplicon reads. Nat. Methods **10**:996–998.

**Estaki, M., Jiang, L., Bokulich, N.A., McDonald, D., González, A., Kosciolek, T., Martino, C., Zhu, Q., Birmingham, A., Vázquez-Baeza, Y., et al.** (2020). QIIME 2 Enables Comprehensive End-to-End Analysis of Diverse Microbiome Data and Comparative Studies with Publicly Available Data. Curr. Protoc. Bioinformatics **70**, e100.

**Fan, D., Ran, L., Hu, J., Ye, X., Xu, D., Li, J., Su, H., Wang, X., Ren, S., and Luo, K.** (2020). miR319a/TCP module and DELLA protein regulate trichome initiation synergistically and improve insect defenses in Populus tomentosa. New Phytol. **227**:867–883.

**Fu, X., Peng, B., Hassani, D., Xie, L., Liu, H., Li, Y., Chen, T., Liu, P., Tang, Y., Li, L., et al.** (2021). AaWRKY9 contributes to light- and jasmonate-mediated to regulate the biosynthesis of artemisinin in Artemisia annua. New Phytol. **231**:1858–1874.

**Gillissen, G., and Becher, E.** (1957). [Reduction of triphenyl tetrazolium chloride (TTC) as a method for determination of antibacterial value]. Arch. Hyg. Bakteriol. **141**:403–420.

**Giraldo, J.P., Landry, M.P., Faltermeier, S.M., McNicholas, T.P., Iverson, N.M., Boghossian, A.A., Reuel, N.F., Hilmer, A.J., Sen, F., Brew, J.A., et al.** (2014). Plant nanobionics approach to augment photosynthesis and biochemical sensing. Nat. Mater. **13**:400–408.

**Graham, I.A., Besser, K., Blumer, S., Branigan, C.A., Czechowski, T., Elias, L., Guterman, I., Harvey, D., Isaac, P.G., Khan, A.M., et al.** (2010). The Genetic Map of *Artemisia annua* L. Identifies Loci Affecting Yield of the Antimalarial Drug Artemisinin. Science **327**:328–331.

**Grice, E.A., Kong, H.H., Conlan, S., Deming, C.B., Davis, J., Young, A.C., NISC Comparative Sequencing Program, Bouffard, G.G., Blakesley, R.W., Murray, R.R., et al.** (2009). Topographical and temporal diversity of the human skin microbiome. Science. **324**:1190–1192.

**Guo, Z., Hao, K., Lv, Z., Yu, L., Bu, Q., Ren, J., Zhang, H., Chen, R., and Zhang, L.** (2022). Profiling of phytohormone-specific microRNAs and characterization of the miR160-ARF1 module involved in glandular trichome development and artemisinin biosynthesis in Artemisia annua. Plant Biotechnol. J. **21**:591–605.

**Gutierrez, T.** (2017). Aerobic hydrocarbon-degrading gammaproteobacteria: Xanthomonadales. In Taxonomy, Genomics and Ecophysiology of Hydrocarbon-Degrading Microbes, T.J. McGenity, ed. (Springer International Publishing), pp. 1–15.

**Hassani, D., Fu, X., Shen, Q., Khalid, M., Rose, J.K.C., and Tang, K.** (2020). Parallel Transcriptional Regulation of Artemisinin and Flavonoid Biosynthesis. Trends Plant Sci. **25**:466–476.

**He, Y., Qian, L., Liu, X., Hu, R., Huang, M., Liu, Y., Chen, G., Losic, D., and Zhu, H.** (2018). Graphene oxide as an antimicrobial agent can extend the vase life of cut flowers. Nano Res. **11**:6010–6022.

**Huang, C., Xia, T., Niu, J., Yang, Y., Lin, S., Wang, X., Yang, G., Mao, L., and Xing, B.** (2018). Transformation of (14) C-Labeled Graphene to (14) CO(2) in the Shoots of a Rice Plant. Angew. Chem., Int. Ed. Engl. **57**:9759–9763.

**Jain, R.G., Fletcher, S.J., Manzie, N., Robinson, K.E., Li, P., Lu, E., Brosnan, C.A., Xu, Z.P., and Mitter, N.** (2022). Foliar application of clay-delivered RNA interference for whitefly control. Nat. Plants **8**:535–548.

**Jiang, X., and Takacs-Vesbach, C.D.** (2017). Microbial community analysis of pH 4 thermal springs in Yellowstone National Park. Extremophiles **21**:135–152.

**Jiang, X., Van Horn, D.J., Okie, J.G., Buelow, H.N., Schwartz, E., Colman, D.R., Feeser, K.L., and Takacs-Vesbach, C.D.** (2022). Limits to the three domains of life: lessons from community assembly along an Antarctic salinity gradient. Extremophiles **26**:15.

**Kah, M., Tufenkji, N., and White, J.C.** (2019). Nano-enabled strategies to enhance crop nutrition and protection. Nat. Nanotechnol. **14**:532–540.

**Kappler, U., Davenport, K., Beatson, S., Lucas, S., Lapidus, A., Copeland, A., Berry, K.W., Glavina Del Rio, T., Hammon, N., Dalin, E., et al.** (2012). Complete genome sequence of the facultatively chemolithoautotrophic and methylotrophic alpha Proteobacterium Starkeya novella type strain (ATCC 8093(T)). Stand. Genomic Sci. **7**:44–58.

**Khan, S., Ali, A., Saifi, M., Saxena, P., Ahlawat, S., and Abdin, M.Z.** (2020). Identification and the potential involvement of miRNAs in the regulation of artemisinin biosynthesis in A. annua. Sci. Rep. **10**, 13614.

**Kung, S.H., Lund, S., Murarka, A., McPhee, D., and Paddon, C.J.** (2018). Approaches and Recent Developments for the Commercial Production of Semi-synthetic Artemisinin. Front. Plant Sci. **9**.

**Lalwani, G., Xing, W., and Sitharaman, B.** (2014). Enzymatic degradation of oxidized and reduced graphene nanoribbons by lignin peroxidase. J. Mater. Chem. B **2**:6354–6362.

**Landry, M.P., and Mitter, N.** (2019). How nanocarriers delivering cargos in plants can change the GMO landscape. Nat. Nanotechnol. **14**:512–514.

**Lawley, B., and Tannock, G.W.** (2017). Analysis of 16S rRNA Gene Amplicon Sequences Using the QIIME Software Package. In Oral Biology: Molecular Techniques and Applications, 2nd Edition, G.J. Seymour, M.P. Cullinan, and N.C.K. Heng, eds., pp. 153–163.

**Li, M., Gao, L., White, J.C., Haynes, C.L., O'Keefe, T.L., Rui, Y., Ullah, S., Guo, Z., Lynch, I., and Zhang, P.** (2023). Nano-enabled strategies to enhance biological nitrogen fixation. Nat. Nanotechnol. **18**:688–691.

**Li, S., Li, J., Du, M., Deng, G., Song, Z., and Han, H.** (2022). Efficient Gene Silencing in Intact Plant Cells Using siRNA Delivered By Functional Graphene Oxide Nanoparticles. Angew. Chem., Int. Ed. Engl. **61**.

**Lian, Y., Liu, W., Shi, R., Zeb, A., Wang, Q., Li, J., Zheng, Z., and Tang, J.** (2022). Effects of polyethylene and polylactic acid microplastics on plant growth and bacterial community in the soil. J. Hazard Mater. **435**, 129057.

**Liao, B., Shen, X., Xiang, L., Guo, S., Chen, S., Meng, Y., Liang, Y., Ding, D., Bai, J., Zhang, D., et al.** (2022). Allele-aware chromosome-level genome assembly of Artemisia annua reveals the correlation between ADS expansion and artemisinin yield. Mol. Plant **15**:1310–1328.

**Liu, H., Li, L., Fu, X., Li, Y., Chen, T., Qin, W., Yan, X., Wu, Z., Xie, L., Kayani, S.L., et al.** (2023). AaMYB108 is the core factor integrating light and jasmonic acid signaling to regulate artemisinin biosynthesis in Artemisia annua. New Phytol. **237**:2224–2237.

**Lu, J., Liu, Y., Zou, X., Zhang, X., Yu, X., Wang, Y., and Si, T.** (2024). Rotational strip peanut/cotton intercropping improves agricultural production through modulating plant growth, root exudates, and soil microbial communities. Agric. Ecosyst. Environ. **359**, 108767.

**Lu, X., Zhang, L., Zhang, F., Jiang, W., Shen, Q., Zhang, L., Lv, Z., Wang, G., and Tang, K.** (2013). AaORA, a trichome-specific AP2/ERF transcription factor of Artemisia annua, is a positive regulator in the artemisinin biosynthetic pathway and in disease resistance to Botrytis cinerea. New Phytol. **198**:1191–1202.

**Ma, D.-M., Wang, Z., Wang, L., Alejos-Gonzales, F., Sun, M.-A., and Xie, D.-Y.** (2015). A Genome-Wide Scenario of Terpene Pathways in Self-pollinated Artemisia annua. Mol. Plant **8**:1580–1598.

**Ma, Y.N., Xu, D.B., Li, L., Zhang, F., Fu, X.Q., Shen, Q., Lyu, X.Y., Wu, Z.K., Pan, Q.F., Shi, P., et al.** (2018). Jasmonate promotes artemisinin biosynthesis by activating the TCP14-ORA complex in Artemisia annua. Sci. Adv. **4**, eaas9357.

**Mitter, N., and Hussey, K.** (2019). Moving policy and regulation forward for nanotechnology applications in agriculture. Nat. Nanotechnol. **14**:508–510.

**Mukherjee, A., Majumdar, S., Servin, A.D., Pagano, L., Dhankher, O.P., and White, J.C.** (2016). Carbon nanomaterials in agriculture: A critical review. Front. Plant Sci. **7**:172.

**Nadali, B.J., Arvind, B., Kamaruzaman, M., and Chan, L.K.** (2014). New cultivation approaches of Artemisia annua L. for a sustainable production of the antimalarial drug artemisinin. J. Med. Plants Res. **8**:441–447.

**Normile, D.** (2015). DRUG DISCOVERY. Nobel for antimalarial drug highlights East-West divide. Science **350**:265.

**Pandey, K., Anas, M., Hicks, V.K., Green, M.J., and Khodakovskaya, M.V.** (2019). Improvement of Commercially Valuable Traits of Industrial Crops by Application of Carbon-based Nanomaterials. Sci. Rep. **9**, 19358.

**Qin, W., Xie, L., Li, Y., Liu, H., Fu, X., Chen, T., Hassani, D., Li, L., Sun, X., and Tang, K.** (2021). An R2R3-MYB Transcription Factor Positively Regulates the Glandular Secretory Trichome Initiation in Artemisia annua L. Front. Plant Sci. **12**, 657156.

**Qu, Y., Wang, J., Ma, Q., Shen, W., Pei, X., You, S., Yin, Q., and Li, X.** (2018). A novel environmental fate of graphene oxide: Biodegradation by a bacterium Labrys sp. WJW to support growth. Water Res. **143**:260–269.

**Quast, C., Pruesse, E., Yilmaz, P., Gerken, J., Schweer, T., Yarza, P., Peplies, J., and Glöckner, F.O.** (2013). The SILVA ribosomal RNA gene database project: improved data processing and web-based tools. Nucleic Acids Res. **41**:D590–D596.

**Segata, N., Izard, J., Waldron, L., Gevers, D., Miropolsky, L., Garrett, W.S., and Huttenhower, C.** (2011). Metagenomic biomarker discovery and explanation. Genome Biol. **12**.

**Shen, Q., Zhang, L., Liao, Z., Wang, S., Yan, T., Shi, P., Liu, M., Fu, X., Pan, Q., Wang, Y., et al.** (2018). The Genome of Artemisia annua Provides Insight into the Evolution of Asteraceae Family and Artemisinin Biosynthesis. Mol. Plant **11**:776–788.

**Shi, P., Fu, X., Shen, Q., Liu, M., Pan, Q., Tang, Y., Jiang, W., Lv, Z., Yan, T., Ma, Y., et al.** (2018). The roles of AaMIXTA1 in regulating the initiation of glandular trichomes and cuticle biosynthesis in Artemisia annua. New Phytol. **217**:261–276.

**Song, Y., Qu, K., Zhao, C., Ren, J., and Qu, X.** (2010). Graphene Oxide: Intrinsic Peroxidase Catalytic Activity and Its Application to Glucose Detection. Adv. Mater. **22**:2206–2210.

**Su, Y., Zhou, X., Meng, H., Xia, T., Liu, H., Rolshausen, P., Roper, C., McLean, J.E., Zhang, Y., Keller, A.A., et al.** (2022). Cost–benefit analysis of nanofertilizers and nanopesticides emphasizes the need to improve the efficiency of nanoformulations for widescale adoption. Nat. Food **3**:1020–1030.

**Tian, S., Guo, R., Zou, X., Zhang, X., Yu, X., Zhan, Y., Ci, D., Wang, M., Wang, Y., and Si, T.** (2019). Priming With the Green Leaf Volatile (Z)-3-Hexeny-1-yl Acetate Enhances Salinity Stress Tolerance in Peanut (Arachis hypogaea L.) Seedlings. Front. Plant Sci. **10**:785.

**Wang, D., and White, J.C.** (2022). Benefit of nano-enabled agrochemicals. Nat. Food **3**:983–984.

**Wang, Q., Garrity, G.M., Tiedje, J.M., and Cole, J.R.** (2007). Naive Bayesian classifier for rapid assignment of rRNA sequences into the new bacterial taxonomy. Appl. Environ. Microbiol. **73**:5261–5267.

**Wang, S., Liu, Y., Wang, X., Xiang, H., Kong, D., Wei, N., Guo, W., and Sun, H.** (2023). Effects of concentration-dependent graphene on maize seedling development and soil nutrients. Sci. Rep. **13**:2650.

**Wang, Z., Yang, Z., and Li, F.** (2019). Updates on molecular mechanisms in the development of branched trichome in Arabidopsis and nonbranched in cotton. Plant Biotechnol. J. **17**:1706–1722.

**Wu, C., Ma, Y., Wang, D., Shan, Y., Song, X., Hu, H., Ren, X., Ma, X., Cui, J., and Ma, Y.** (2022). Integrated microbiology and metabolomics analysis reveal plastic mulch film residue affects soil microorganisms and their metabolic functions. J. Hazard Mater. **423**, 127258.

**Xiao, L., Tan, H., and Zhang, L.** (2016). Artemisia annua glandular secretory trichomes: the biofactory of antimalarial agent artemisinin. Sci. Bull. **61**:26–36.

**Xie, L., Yan, T., Li, L., Chen, M., Hassani, D., Li, Y., Qin, W., Liu, H., Chen, T., Fu, X., et al.** (2021a). An HD-ZIP-MYB complex regulates glandular secretory trichome initiation in Artemisia annua. New Phytol. **231**:2050–2064.

**Xie, L., Yan, T., Li, L., Chen, M., Ma, Y., Hao, X., Fu, X., Shen, Q., Huang, Y., Qin, W., et al.** (2021b). The WRKY transcription factor AaGSW2 promotes glandular trichome initiation in Artemisia annua. J. Exp. Bot. **72**:1691–1701.

**Xie, W., Zhang, K., Wang, X.Y., Zou, X.X., Zhang, X.J., Yu, X.N., Wang, Y.F., and Si, T.** (2022). Peanut and cotton intercropping increases productivity and economic returns through regulating plant nutrient accumulation and soil microbial communities. BMC Plant Biol. **22**.

**Xing, L., Zhu, M., Luan, M., Zhang, M., Jin, L., Liu, Y., Zou, J., Wang, L., and Xu, M.** (2022). miR169q and NUCLEAR FACTOR YA8 enhance salt tolerance by activating PEROXIDASE1 expression in response to ROS. Plant Physiol. **188**:608–623.

**Xue, X.Y., Zhao, B., Chao, L.M., Chen, D.Y., Cui, W.R., Mao, Y.B., Wang, L.J., and Chen, X.Y.** (2014). Interaction between two timing microRNAs controls trichome distribution in Arabidopsis. PLoS Genet. **10**, e1004266.

**Yan, T., Li, L., Xie, L., Chen, M., Shen, Q., Pan, Q., Fu, X., Shi, P., Tang, Y., Huang, H., et al.** (2018). A novel HD-ZIP IV/MIXTA complex promotes glandular trichome initiation and cuticle development in Artemisia annua. New Phytol. **218**:567–578.

**Yan, T., Chen, M., Shen, Q., Li, L., Fu, X., Pan, Q., Tang, Y., Shi, P., Lv, Z., Jiang, W., et al.** (2017). HOMEODOMAIN PROTEIN 1 is required for jasmonate-mediated glandular trichome initiation in Artemisia annua. New Phytol. **213**:1145–1155.

**Yu, N., Cai, W.J., Wang, S., Shan, C.M., Wang, L.J., and Chen, X.Y.** (2010). Temporal control of trichome distribution by microRNA156-targeted SPL genes in Arabidopsis thaliana. Plant Cell **22**:2322–2335.

**Yu, Y., Li, X., Feng, Z., Xiao, M., Ge, T., Li, Y., and Yao, H.** (2022). Polyethylene microplastics alter the microbial functional gene abundances and increase nitrous oxide emissions from paddy soils. J. Hazard Mater. **432**, 128721.

**Yuan, M., Shu, G., Zhou, J., He, P., Xiang, L., Yang, C., Chen, M., Liao, Z., and Zhang, F.** (2023). AabHLH113 integrates jasmonic acid and abscisic acid signaling to positively regulate artemisinin biosynthesis in Artemisia annua. New Phytol. **237**:885–899.

**Zhang, F., Fu, X., Lv, Z., Lu, X., Shen, Q., Zhang, L., Zhu, M., Wang, G., Sun, X., Liao, Z., et al.** (2015). A basic leucine zipper transcription

factor, AabZIP1, connects abscisic acid signaling with artemisinin biosynthesis in Artemisia annua. Mol. Plant **8**:163–175.

**Zhang, X., Cao, H., Wang, J., Li, F., and Zhao, J.** (2022). Graphene oxide exhibits antifungal activity against *Bipolaris sorokiniana* in vitro and in vivo. Microorganisms **10**, 1994.

**Zhao, B., Cao, J.F., Hu, G.J., Chen, Z.W., Wang, L.Y., Shangguan, X.X., Wang, L.J., Mao, Y.B., Zhang, T.Z., Wendel, J.F., et al.** (2018). Core cis-element variation confers subgenome-biased expression of a transcription factor that functions in cotton fiber elongation. New Phytol. **218**:1061–1075.

**Zhao, L., Bai, T., Wei, H., Gardea-Torresdey, J.L., Keller, A., and White, J.C.** (2022). Nanobiotechnology-based strategies for enhanced crop stress resilience. Nat. Food **3**:829–836.

**Zheng, H., Fu, X., Shao, J., Tang, Y., Yu, M., Li, L., Huang, L., and Tang, K.** (2023). Transcriptional regulatory network of high-value active ingredients in medicinal plants. Trends Plant Sci. **28**:429–446.

**Zhu, J., Liu, S., Wang, H., Wang, D., Zhu, Y., Wang, J., He, Y., Zheng, Q., and Zhan, X.** (2022). Microplastic particles alter wheat rhizosphere soil microbial community composition and function. J. Hazard Mater. **436**, 129176.

Plant Communications, Volume *5*

# Supplemental information

# Graphene enhances artemisinin production in the traditional medicinal plant *Artemisia annua* via dynamic physiological processes and miRNA regulation

**Junfeng Cao, Zhiwen Chen, Luyao Wang, Ning Yan, Jialing Lin, Lipan Hou, Yongyan Zhao, Chaochen Huang, Tingting Wen, Chenyi Li, Saeed ur Rahman, Zehui Liu, Jun Qiao, Jianguo Zhao, Jie Wang, Yannan Shi, Wei Qin, Tong Si, Yuliang Wang, and Kexuan Tang**

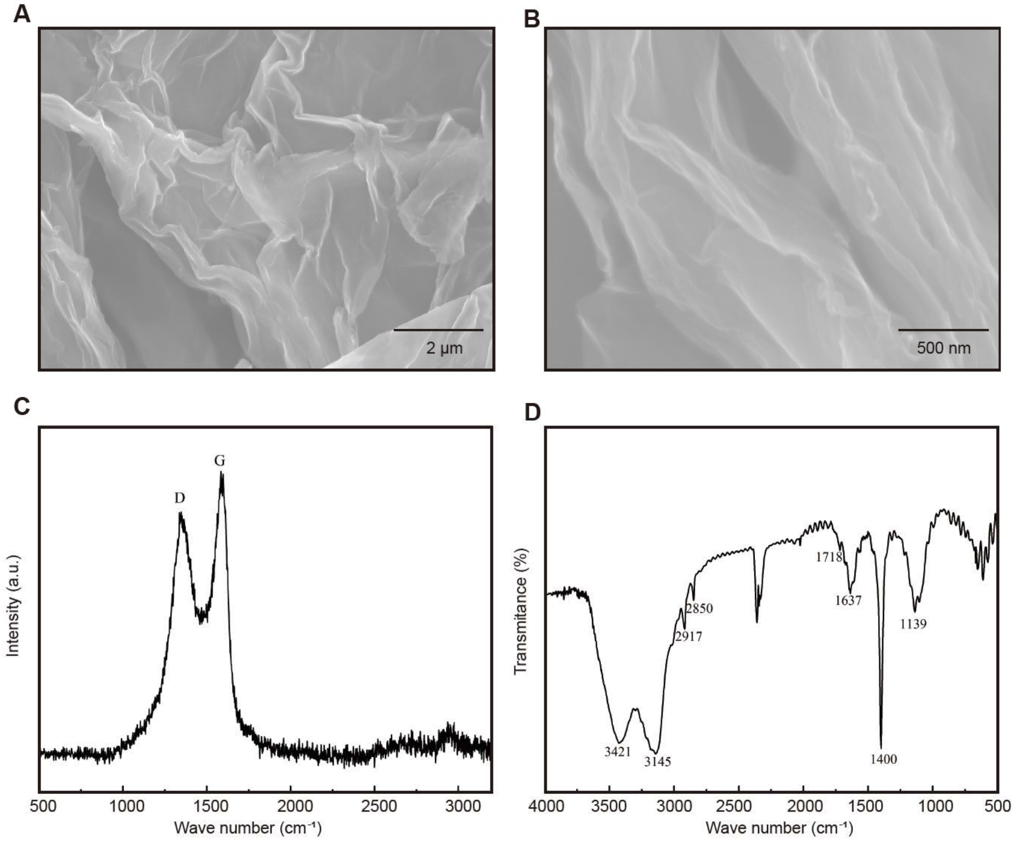


**Supplementary Figure 1 Characterization of graphene.**

**(A and B)** SEM image, **(C)** Raman spectrum, and **(D)** Fourier transform infrared (FT-IR) spectrum.

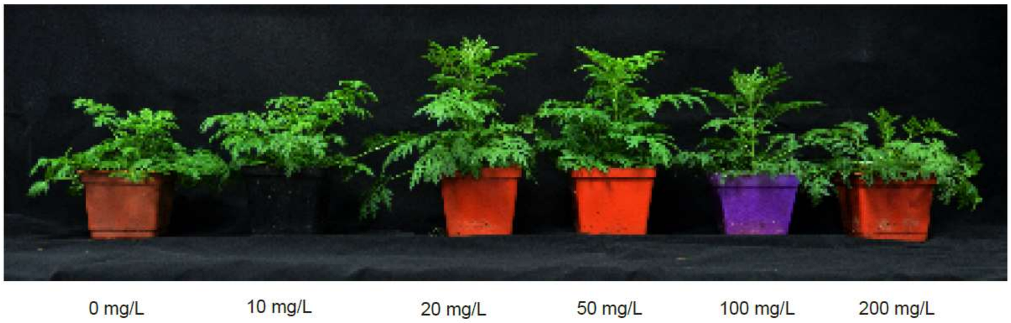


**Supplementary Figure 2 Phenotypes of *A. annua* treated with different concentration of graphene.**

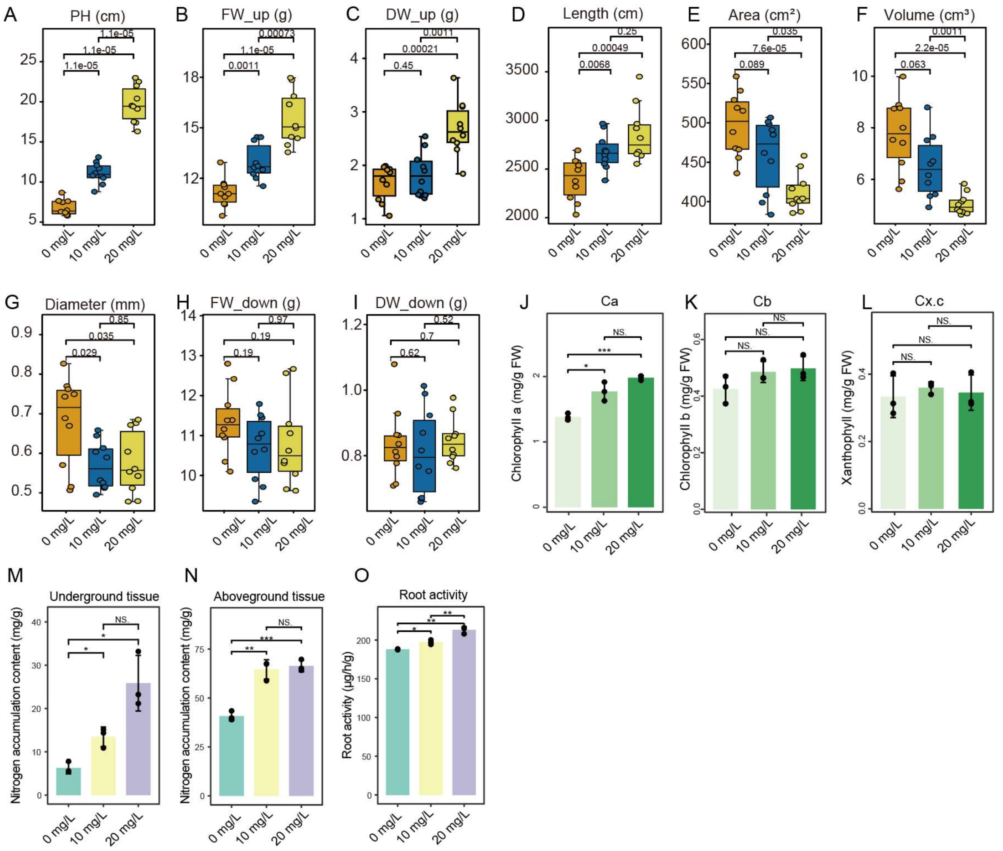


**Supplementary Figure 3 Statistics of phenotypes and physiological data of *A. annua* treated with graphene.**

**(A-C)** statistics analysis of overground parts seedlings exposed to different graphene concentrations shown in **Figure 1A**. **(A)** Plant height (PH). **(B)** Fresh weight of shoots (FW_up). **(c)** Dry weight of shoots (DW_up), mean ± SD, n = 10, Student's *t*-test.

**(D-I)** Statistics of root phenotypes generated from **Figure 1B**, including total root length **(D)**, total root surface area **(E)**, total root volume **(F)** and root average diameter **(G)**. **(H)** Fresh weight of roots (FW_down). **(I)** Dry weight of roots (DW_down), mean ± SD, n = 10, Student's *t*-test.

**(J-L)** Statistics of the photosynthetic pigments **(J)** chlorophyll a, **(K)** chlorophyll b and **(L)** xanthophyll.

**(M-O)** Statistics of nitrogen absorption and assimilation in **Figure 1**. **(M and N)** total nitrogen accumulated in the underground **(M)** and aboveground **(N)** parts of the seedlings. **(O)** root activity measured by TTC.

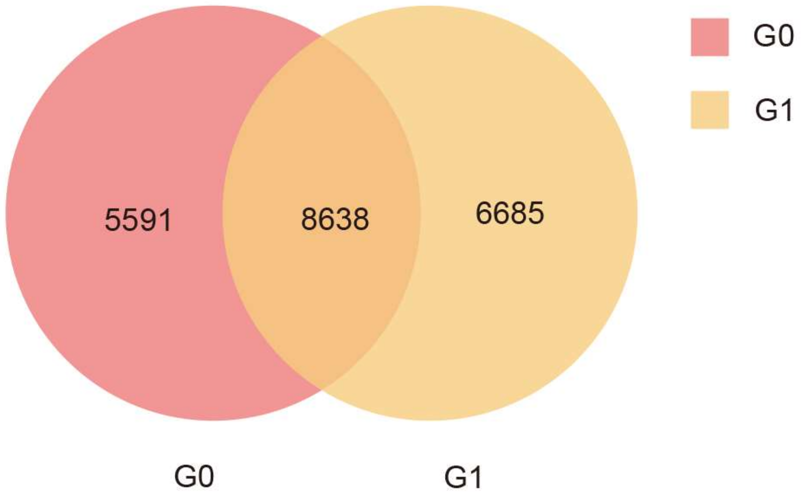


**Supplementary Figure 4 Differentially expressed genes of leaf between CK and graphene treatment groups.** G0, 0 vs 10 mg/L; G1, 0 vs 20 mg/L.

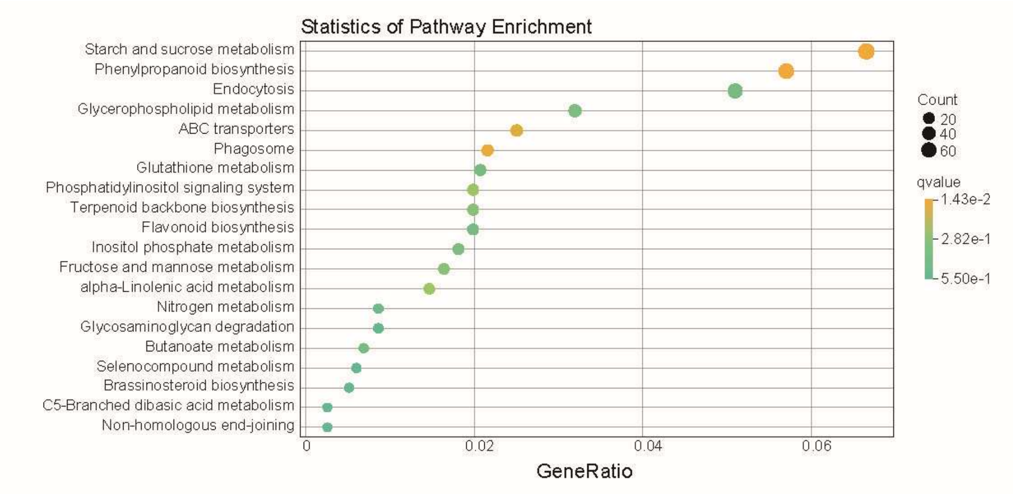


**Supplementary Figure 5 Top 20 KEGG pathway analyses of the enriched differentially expressed genes after graphene treatment.**

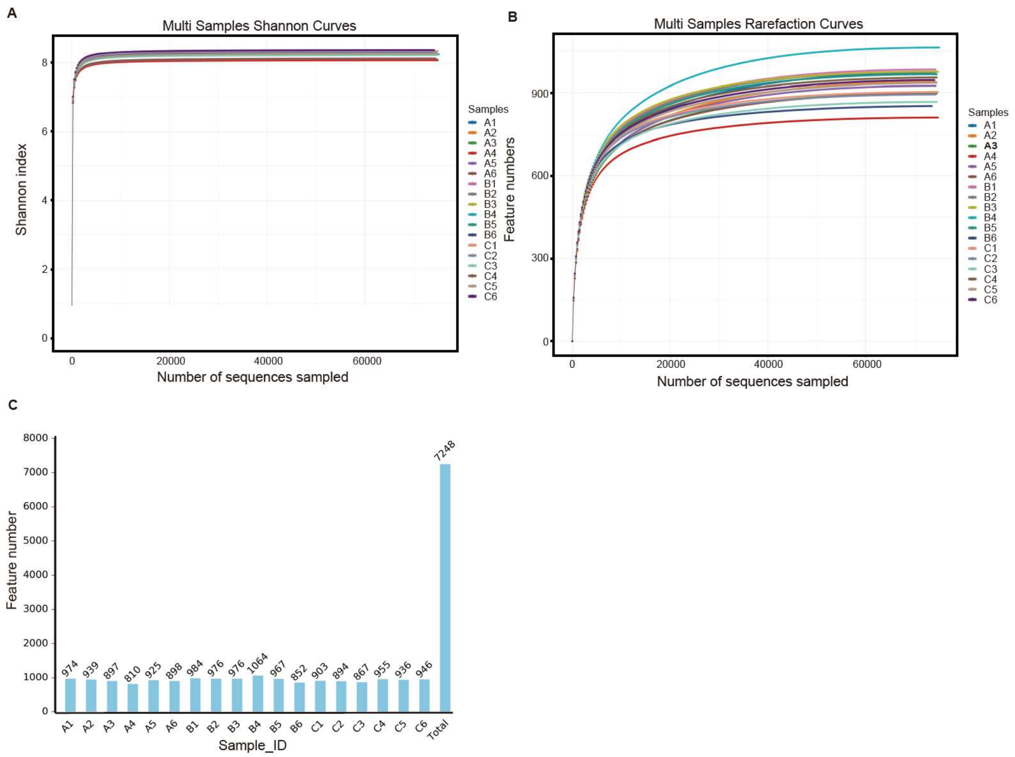


**Supplementary Figure 6 Sequencing statistics and basic OTU analyses.**

**(A)** bacterial 16S DNA Shannon-Wiener curve, **(B)** bacterial 16S DNA Rarefaction curve,

**(C)** bacterial species OTUs number. Group A: 0 mg/L, Group B: 10 mg/L, Group C: 20 mg/L

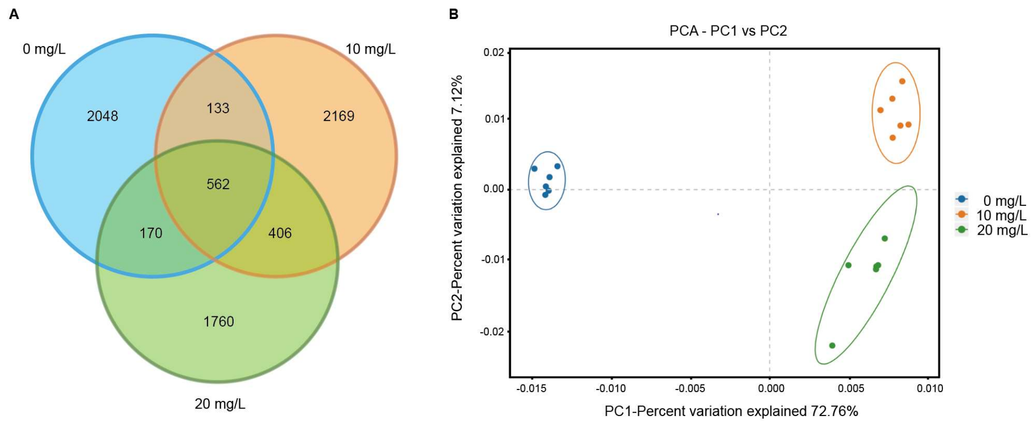


**Supplementary Figure 7 Rhizosphere microbial changes between CK and graphene treatment groups.**

**(A)** Venn diagrams of the bacterial OTUs number, **(B)** Principal Component Analysis (PCA) of the rhizosphere bacterial community.

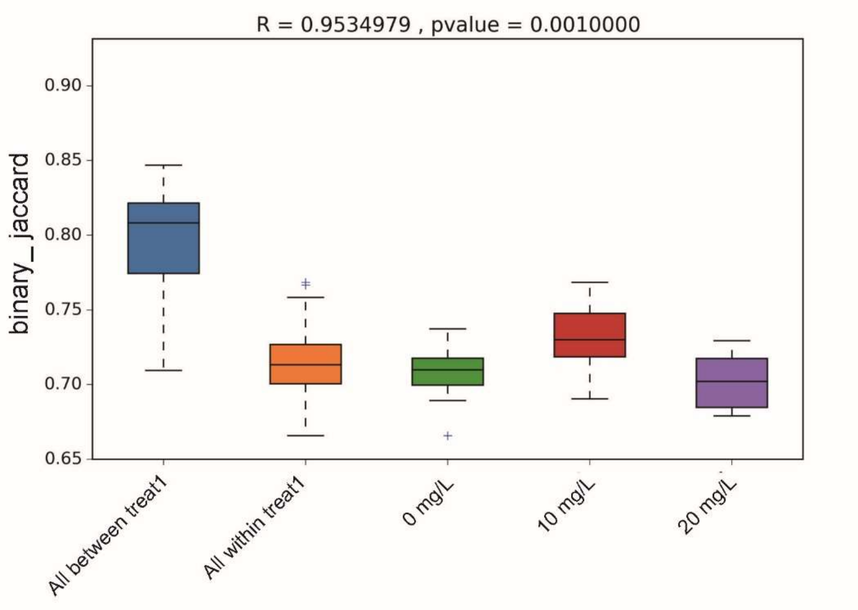


**Supplementary Figure 8 ANOSIM analysis boxplot.**

The R-value (between -1 and 1) is greater than 0, indicating that the difference between the groups is significant. The credibility of the statistical analysis is expressed by *P*-value, and $P$ <0.05 indicates significance.

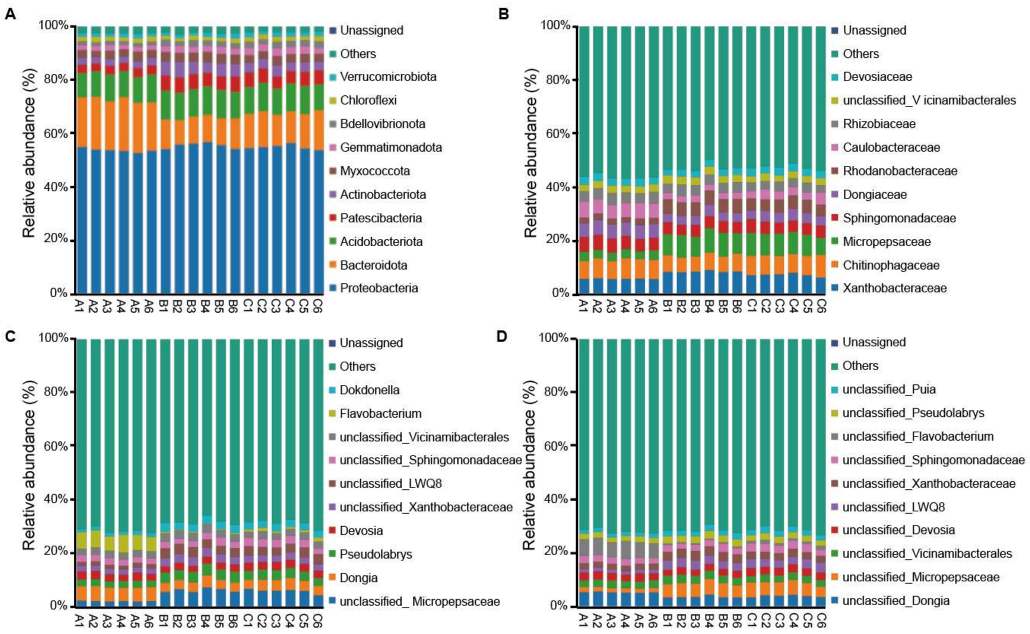


**Supplementary Figure 9** Changes in rhizosphere bacterial community diversity between the control (A samples) and graphene treatment groups (B samples 10 mg/L, C samples 20 mg/L). Proportion (%) of major bacteria at the phylum **(A)**, family **(B)**, genus **(C)** and species **(D)** levels.

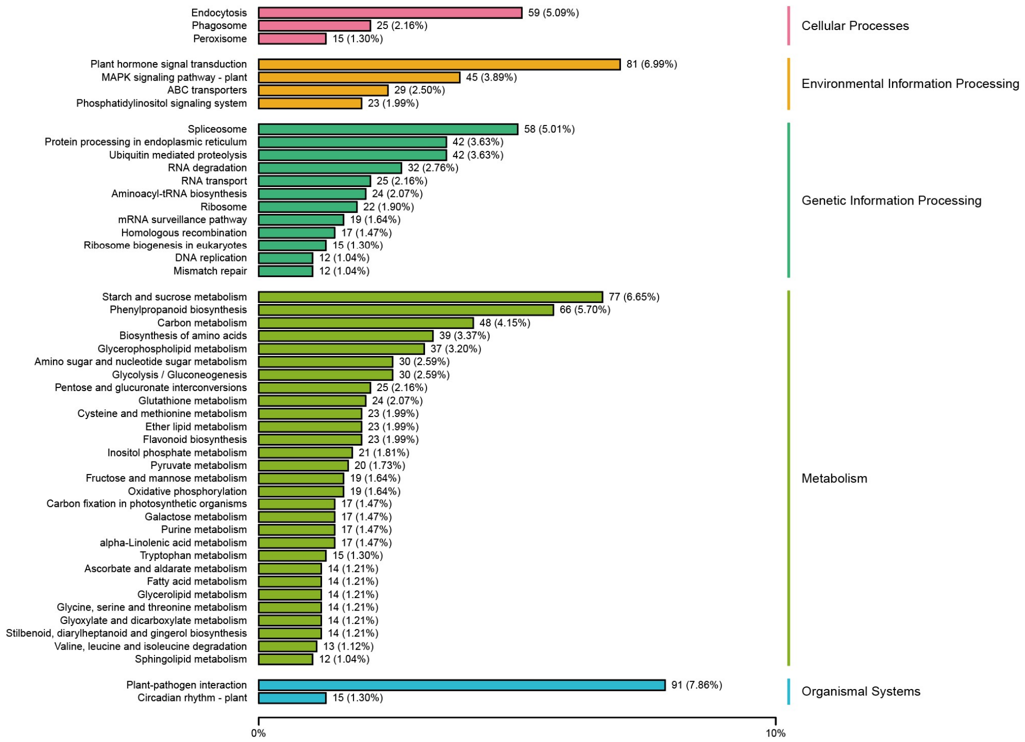


**Supplementary Figure 10 KEGG classification analyses of the enriched differentially expressed genes.**

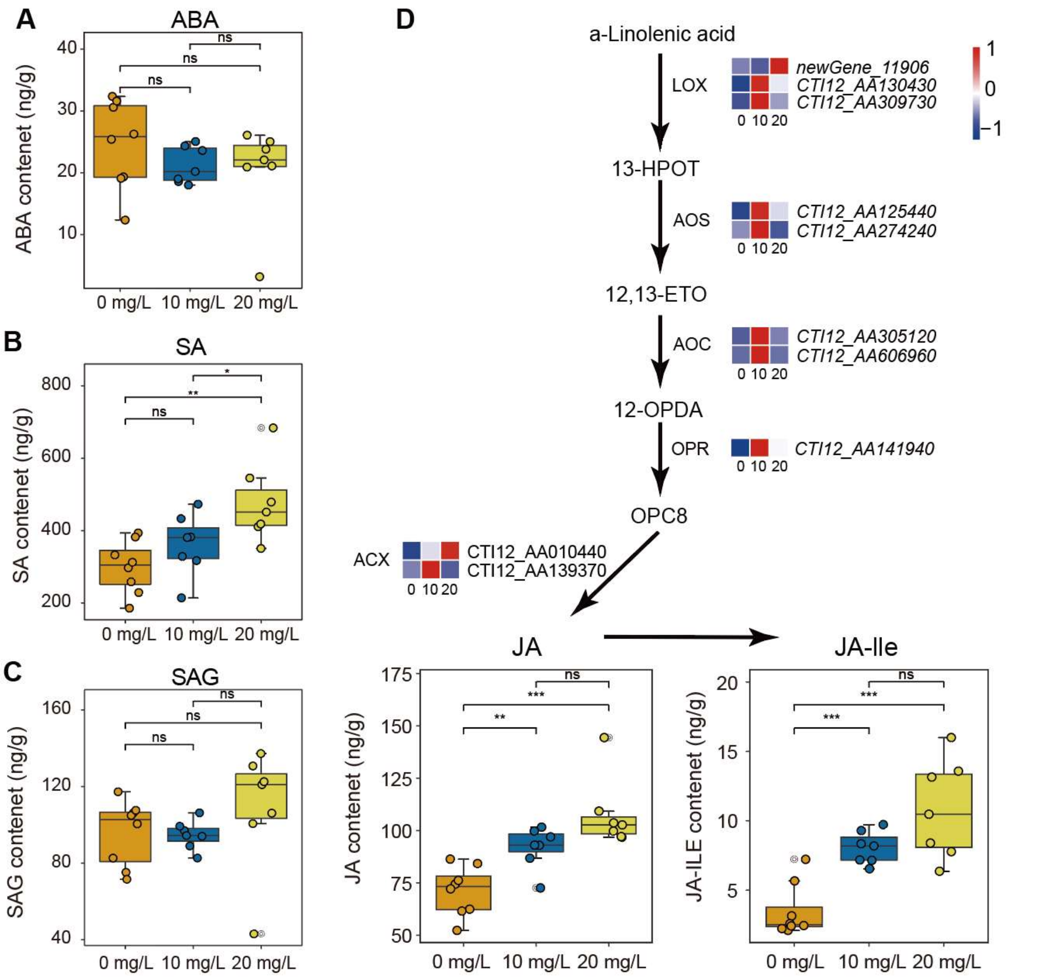


**Supplementary Figure 11 Graphene promotes the biosynthesis of stress-related phytohormones**

**(A-D)** The contents of endogenous ABA **(A)**, SA **(B)** and its derivative SAG **(C)** with graphene treatments. **(D)** The enhanced biosynthetic pathway and products of JA, the essential regulator for artemisinin. The expression level of enzymes located in JA pathway is presented by the heatmap according to the RNA-seq. The contents of endogenous JA and JA-IIE are under the pathway, mean ± SD, n = 7, *$P < 0.05$, **$P < 0.01$, Student's *t*-test.

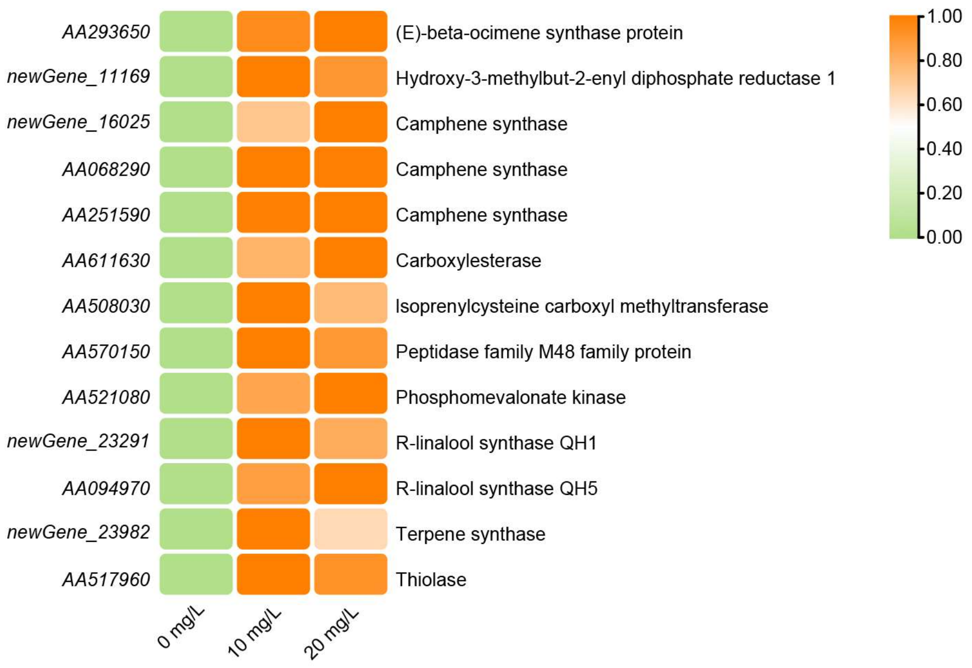


**Supplementary Figure 12 Heatmap of DEGs involved in terpenoid backbone biosynthesis.**

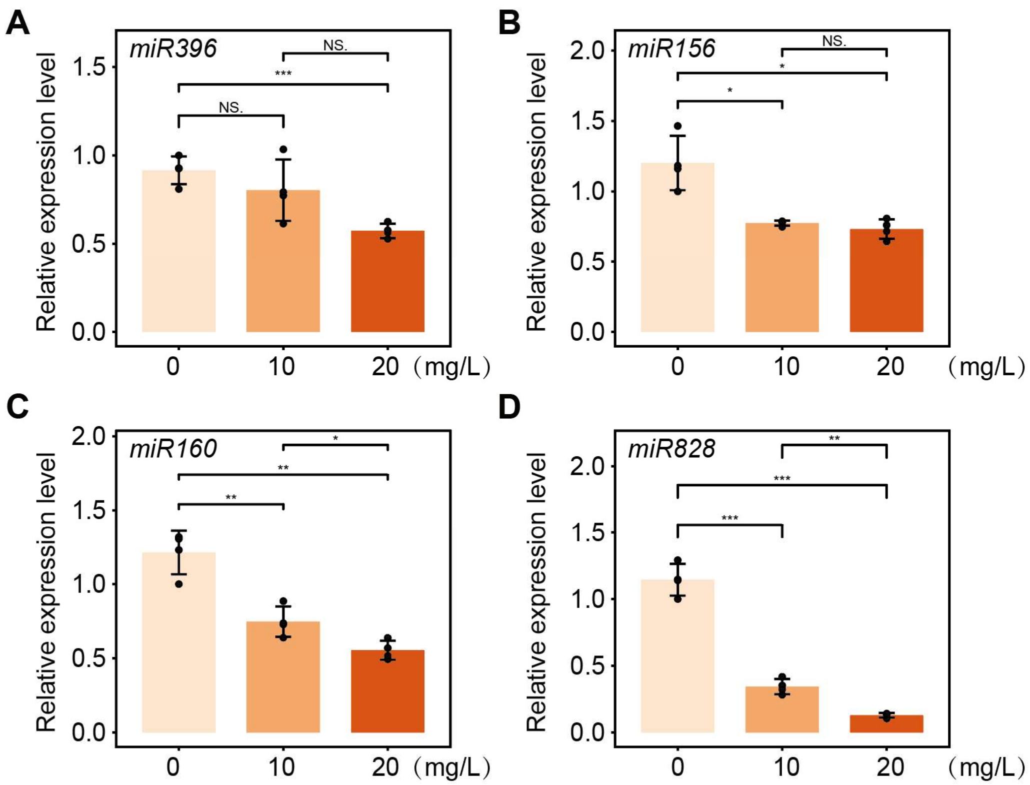


**Supplementary Figure 13 Graphene decreases the expression of miRNAs.**

**(A-D)** Relative expression of mature miRNAs in *A. annua*, the level at 0 mg/L was set to one (means of triplicates ± s.d.), U6 served as internal reference. *$P < 0.05$, **$P < 0.01$, ***$P < 0.001$, Student's *t*-test

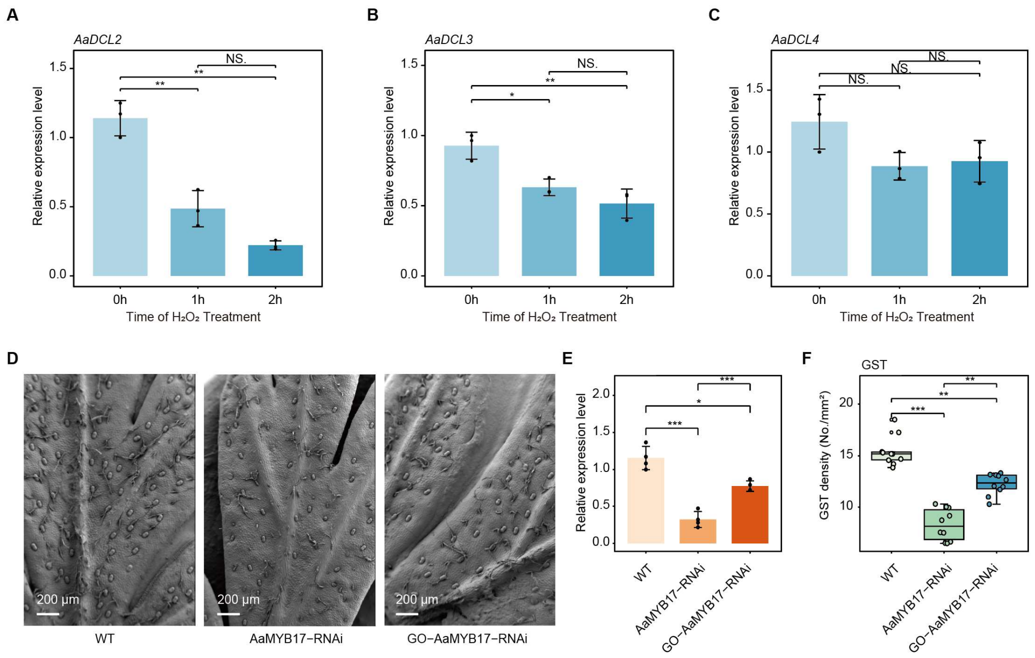


**Supplementary Figure 14 Graphene blocks the siRNA biogenesis to rescue the RNAi effect through dicers.**

**(A-C)** Relative expression of Dicers in *A. annua* treated with 1 mM $H_2O_2$. The level at 0 h (untreated) was set to one (means of triplicates ± s.d.). **P* < 0.05, ***P* < 0.01, Student's *t*-test.

**(H)** Image of GSTs on leaves from wild type (WT), AaMYB17 RNAi lines and 20 mg/L graphene treated AaMYB17 RNAi lines (GPE-AaMYB17-RNAi). **(I)** Relative expression of AaMYB17 in leaves as shown in **(H)** (mean ± s.d., n=4, **P* < 0.05, ****P* < 0.001, Student's *t*-test). **(J)** GST densities of the samples from **(H)** (mean ± s.d., n=10, ***P* < 0.01, ****P* < 0.001, Student's *t*-test).

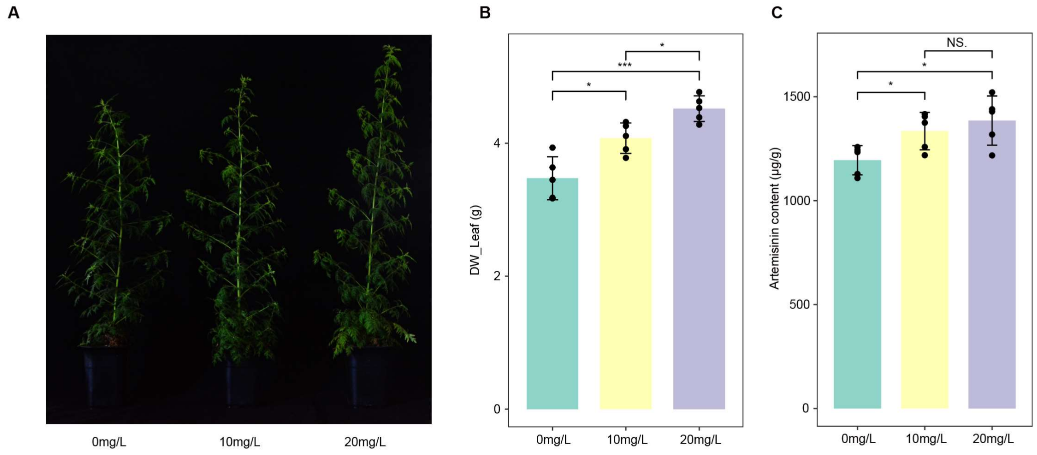


## Supplementary Figure 15 Graphene improves artemisinin yield

**(A)** Image of 4-month-old plants treated with graphene. **(B)** the dry weight of total leaves. **(C)** the artemisinin content per unit weight of total leaves from **(A)** mean ± s.d., n=5, $*P < 0.05$, $***P < 0.001$, Student's *t*-test

**Supplementary Table 1. Statistics of bacteria 16S DNA sequencing data.**

| Sample ID | Raw Reads | Clean Reads | Denoised Reads | Merged Reads | Non-chimeric Reads | Effective Ratio (%) |
|---|---|---|---|---|---|---|
| A0-1 | 80,009 | 79,674 | 77,884 | 75,467 | 74,381 | 92.97% |
| A0-2 | 80,256 | 79,889 | 77,986 | 75,411 | 74,277 | 92.55% |
| A0-3 | 80,157 | 79,820 | 78,166 | 75,791 | 74,739 | 93.24% |
| A0-4 | 80,065 | 79,664 | 78,064 | 75,938 | 74,818 | 93.45% |
| A0-5 | 80,070 | 79,706 | 77,881 | 75,447 | 74,300 | 92.79% |
| A0-6 | 80,020 | 79,619 | 77,866 | 75,471 | 74,290 | 92.84% |
| B10-1 | 80,246 | 79,921 | 78,076 | 75,348 | 74,485 | 92.82% |
| B10-2 | 80,053 | 79,729 | 78,083 | 75,622 | 74,674 | 93.28% |
| B10-3 | 79,946 | 79,614 | 77,966 | 75,778 | 74,778 | 93.54% |
| B10-4 | 80,023 | 79,681 | 78,051 | 76,124 | 75,233 | 94.01% |
| B10-5 | 79,730 | 79,401 | 77,784 | 75,463 | 74,686 | 93.67% |
| B10-6 | 80,161 | 79,782 | 77,834 | 75,446 | 73,645 | 91.87% |
| C20-1 | 79,973 | 79,616 | 77,833 | 75,725 | 74,901 | 93.66% |
| C20-2 | 79,994 | 79,643 | 77,823 | 75,221 | 74,317 | 92.90% |
| C20-3 | 80,107 | 79,772 | 78,009 | 75,643 | 74,642 | 93.18% |
| C20-4 | 79,987 | 79,654 | 77,912 | 75,638 | 74,684 | 93.37% |
| C20-5 | 80,118 | 79,761 | 77,984 | 75,518 | 74,720 | 93.26% |
| C20-6 | 80,085 | 79,728 | 77,794 | 75,512 | 74,204 | 92.66% |

Group A: 0 mg/L, Group B: 10 mg/L, Group C: 20 mg/L

**Supplementary Table 2. Statistics of the bacterial OTUs number at different taxonomy levels: kingdom, phylum, class, order, family, genus and species.**

| Sample | Kingdom | Phylum | Class | Order | Family | Genus | Species |
|---|---|---|---|---|---|---|---|
| A0-1 | 2 | 24 | 47 | 114 | 201 | 294 | 339 |
| A0-2 | 1 | 22 | 44 | 108 | 191 | 288 | 331 |
| A0-3 | 1 | 21 | 41 | 105 | 188 | 276 | 314 |
| A0-4 | 2 | 22 | 44 | 110 | 189 | 282 | 317 |
| A0-5 | 1 | 21 | 45 | 115 | 196 | 283 | 327 |
| A0-6 | 2 | 22 | 42 | 105 | 185 | 281 | 323 |
| B10-1 | 1 | 22 | 46 | 112 | 200 | 289 | 329 |
| B10-2 | 1 | 23 | 44 | 114 | 199 | 298 | 330 |
| B10-3 | 1 | 23 | 46 | 115 | 207 | 301 | 333 |
| B10-4 | 1 | 23 | 48 | 113 | 193 | 294 | 335 |
| B10-5 | 1 | 23 | 46 | 117 | 206 | 295 | 323 |
| B10-6 | 1 | 24 | 49 | 118 | 200 | 290 | 318 |
| C20-1 | 1 | 21 | 45 | 113 | 201 | 296 | 324 |
| C20-2 | 1 | 21 | 43 | 108 | 195 | 286 | 318 |
| C20-3 | 1 | 21 | 43 | 112 | 195 | 289 | 313 |
| C20-4 | 1 | 24 | 50 | 119 | 206 | 303 | 341 |
| C20-5 | 1 | 20 | 43 | 107 | 194 | 288 | 314 |
| C20-6 | 1 | 19 | 42 | 110 | 194 | 293 | 327 |
| Total | 2 | 29 | 69 | 167 | 317 | 527 | 633 |

Group A: 0 mg/L, Group B: 10 mg/L, Group C: 20 mg/L

**Supplementary Table 3. Major primers used in this investigation**

| Primer ID | Sequence | Purpose |
|---|---|---|
| ADS-RT-F | AATGGGCAAATGAGGGACAC | qRT-PCR |
| ADS-RT-R | TTTCAAGGCTCGATGAACTATG | qRT-PCR |
| CYP71AV1-RT-F | CGAGACTTTAACTGGTGAGATTGT | qRT-PCR |
| CYP71AV1-RT-R | CGAAGCGACTGAAATGACTTTACT | qRT-PCR |
| DBR2-RT-F | GCGGTGGTTACACTAGAGAACTT | qRT-PCR |
| DBR2-RT-R | ATAATCAAAACTAGAGGAGTGACCC | qRT-PCR |
| ALDH1-RT-F | GGACTTGCCTCAGGTGTAT | qRT-PCR |
| ALDH1-RT-R | GTGCCTCTAATCCTTGTTC | qRT-PCR |
| AaMYB17-RT-F | TCATTACTACTCCCATCACCCAAC | qRT-PCR |
| AaMYB17-RT-R | CATCGCTCCTTCTGGCACAT | qRT-PCR |
| AaGSW2-RT-F | TTCCTCATCTTCAACTTCACCAC | qRT-PCR |
| AaGSW2-RT-R | CGCTACTCGTGGGAGAAACAT | qRT-PCR |
| AaHD1-RT-F | GCTTGATCCTCACTGCGGTAT | qRT-PCR |
| AaHD1-RT-R | TACGTTCTGGGACGAGTTGCT | qRT-PCR |
| AaSPL9-RT-F | TGGAACTCTGTTATCTGCCCGC | qRT-PCR |
| AaSPL9--RT-R | ATCCTGAACTCCGCTAAAACCTGT | qRT-PCR |
| actin-RT-F | CCAGGCTGTTCAGTCTCTGTAT | qRT-PCR |
| actin-RT-R | CGCTCGGTAAGGATCTTCATCA | qRT-PCR |
| miR828 | TCTTGCTCAAATGAGTATTCTA | microRNA qRT-PCR |
| mir160 | TATGAGGAGCCATGCATGTAT | microRNA qRT-PCR |
| mir156 | TGACAGAAGAGAGTGAGCAC | microRNA qRT-PCR |
| mir396 | TTCCACAGCTTTCTTGAACTG | microRNA qRT-PCR |
| U6 | TGTATCGTTCCAATTTTATCGGATGT | microRNA qRT-PCR |
